# VLDLR disturbs quiescence of breast cancer stem cells in a ligand-independent function

**DOI:** 10.3389/fonc.2022.887035

**Published:** 2022-12-07

**Authors:** Mengying Yang, Yajing Zhan, Zhijie Hou, Chunli Wang, Wenjun Fan, Tao Guo, Zhuoshi Li, Lei Fang, Shasha Lv, Sisi Li, Chundong Gu, Mingliang Ye, Hongqiang Qin, Quentin Liu, Xiaonan Cui

**Affiliations:** ^1^ The First Affiliated Hospital, Dalian Medical University, Dalian, China; ^2^ Institute of Cancer Stem Cell, Cancer Center, Dalian Medical University, Dalian, China; ^3^ State Key Laboratory of Oncology in South China, Cancer Center, Sun Yat-sen University, Guangzhou, China; ^4^ Department of Pathology, Harbin Medical University Cancer Hospital, Harbin, China; ^5^ Chinese Academy of Sciences (CAS) Key Laboratory of Separation Science for Analytical Chemistry, Dalian Institute of Chemical Physics, Chinese Academy of Science, Dalian, China

**Keywords:** VLDLR, breast cancer, cancer stem cell, quiescence, ligand-independent function

## Abstract

Breast cancer stem cells are responsible for cancer initiation, progression, and drug resistance. However, effective targeting strategies against the cell subpopulation are still limited. Here, we unveil two splice variants of very-low-density lipoprotein receptor, VLDLR-I and -II, which are highly expressed in breast cancer stem cells. In breast cancer cells, VLDLR silencing suppresses sphere formation abilities *in vitro* and tumor growth *in vivo*. We find that VLDLR knockdown induces transition from self-renewal to quiescence. Surprisingly, ligand-binding activity is not involved in the cancer-promoting functions of VLDLR-I and -II. Proteomic analysis reveals that citrate cycle and ribosome biogenesis-related proteins are upregulated in VLDLR-I and -II overexpressed cells, suggesting that VLDLR dysregulation is associated with metabolic and anabolic regulation. Moreover, high expression of VLDLR in breast cancer tissues correlates with poor prognosis of patients. Collectively, these findings indicate that VLDLR may be an important therapeutic target for breast cancer treatment.

## Introduction

Breast cancer has been the most common female malignancy and the second leading cause of cancer-related death among women worldwide (1). Even recent advances in diagnosis and treatment have significantly reduced breast cancer mortality (1); the survival rate remains to be improved. Breast cancer stem cells (BCSCs), defined by their ability to self-renew and form a heterogeneous tumor, are responsible for cancer initiation, progression, metastasis, and therapeutic resistance (2). Therefore, targeting BCSCs is one of the key therapeutic strategies for cancer patients.

Very-low-density lipoprotein receptor (VLDLR), a member of the low-density lipoprotein receptor (LDLR) superfamily (3), consists of two subtypes because of alternative splicing, namely the full-length VLDLR (VLDLR-I) and type II VLDLR (VLDLR-II), which lacks the O-linked sugar domain encoded by the 16th exon (4). The distribution of these two VLDLR subtypes presents obvious tissue specificity. VLDLR-I is the major transcript in fatty acid-active tissues, such as the heart and skeletal muscles, in which free fatty acids are important oxidative fuels. However, VLDLR-II predominates in the non-muscular tissues, including the kidney, spleen, adrenal gland, lung, brain, testis, uterus, and ovary (4). VLDLR is originally considered to specifically bind to VLDL and play important roles in lipid metabolism. However, recent studies have found that VLDLR affects many cellular functions due to its ability to bind numerous ligands besides lipoprotein, including lipoprotein lipase (LPL) (5), receptor-associated protein (RAP) (6), thrombospondin-1 (7), urokinase plasminogen activator/plasminogen activator inhibitor-1 complex (uPA/PAI-1) (8), and several other proteinase/serpin complexes (9). Furthermore, binding of Reelin to VLDLR induces tyrosine phosphorylation of Disable-1 (Dab-1), which is essential for neuron migration during brain development (10). This suggests that VLDLR is involved in extracellular signal transduction.

Abnormal VLDLR expression has been associated with the pathogenesis of various cancers (11–13). VLDLR-II has been reported as the predominantly expressed variant in cancer tissues, such as breast and gastric cancer (14, 15). Moreover, VLDLR-II overexpression promoted, while VLDLR-I transfection reduced, cancer cell proliferation and migration in a gastric adenocarcinoma cell line (16), indicating the controversial effects of two VLDLR subtypes. Some studies revealed that VLDLR promotes the growth of cancer cells by binding to the uPA/PAI-1 complex (12), or by regulating the stability of β-catenin (16). Interestingly, recent studies have shown that the expression of VLDLR-II is obviously high in poorly differentiated adenocarcinomas (17), implying that VLDLR-II expression may be associated with cancer cell differentiation. Though the critical role of VLDLR in tumor development has been reported in various cancer types, the function of VLDLR has not been investigated in BCSCs.

In the present study, we found that VLDLR was remarkably upregulated in BCSCs compared to non-BCSCs. VLDLR silencing induced transition to quiescence of breast cancer cells in a ligand-independent manner, blunting cell proliferation *in vitro* and *in vivo*. Moreover, high expression of VLDLR was associated with poor prognosis of patients with breast cancer. Collectively, our findings provided convincing evidences that VLDLR can serve as a promising target for breast cancer therapy.

## Materials and methods

### Cell culture

Human breast cancer cell lines (MDA-MB-231, SK-BR-3, and MCF7) and the human embryonic kidney HEK-293T cell line were obtained from the American Type Culture Collection (ATCC). These cell lines were authenticated at ATCC before purchase by their standard short tandem repeat DNA-typing methodology. MDA-MB-231, SK-BR-3, and HEK-293T cells were maintained in Dulbecco’s modified Eagle’s medium (DMEM, Gibco) containing 10% fetal bovine serum (FBS, Gibco). MCF7 cells were cultured in Minimum Essential Media (MEM, ATCC) containing 10% FBS and 0.01 mg/ml human recombinant insulin (Sigma-Aldrich). All cells were incubated at 37°C in a humidified 5% CO_2_ atmosphere. Lipid-depleted serum (LDS) were purchased from MACGENE (Catalog. CS021).

### Plasmid construction

pLVX-flag was a gift from Dr. Tao Guo (The First Affiliated Hospital, Dalian Medical University, Dalian, China). pLVX-mVenus-p27K^-^ was a gift from Dr. Bing Liu (Institute of Cancer Stem Cell, Cancer Center, Dalian Medical University, Dalian, China). VLDLR-I, VLDLR-II, LDLR, and RAP fragments were cloned from human genome cDNA. The primers were as follows: VLDLR-I/II: Forward, 5′-CTACCGGACTCAGATGCCACCATGGGCACGTCCGCGCTCT; Reverse, 5′-TACCCGGTAGAATTATCTAGATCAAGCTAGATCATCATCT. LDLR: Forward, 5′-CCGCGGCCGCGCCACCATGGGGCCCTGGGGCTGGAA; Reverse, 5′-TCCATATGTCACGCCACGTCATCCTCCA. RAP: Forward, 5′-GATCTGGTTCCGCGTGGATCCATGGCGCCGCGGAGGGTCA; Reverse, 5′-GTCACGATGCGGCCGCTCGAGTCAGAGTTCGTTGTGCCGA. DsRed fragment was replaced by the VLDLR-I/II fragment in the pLVX-DsRed-Monomer-N1 vector (Clontech). The LDLR fragment was ligated into the pLVX-TRE3G vector (Clontech). The RAP fragment was recombinated into the pGEX-4T-1 vector (Clontech). The shRNAs specifically targeting VLDLR were cloned into the pLKO-Tet-On-shNC vector. The primers were as follows: shVLDLR-1: Forward, 5′-CCGGGCACAGATGATGATCTAGCTTCTCGAGAAGCTAGATCATCATCTGTGCTTTTTG; Reverse, 5′-AATTCAAAAAGCACAGATGATGATCTAGCTTCTCGAGAAGCTAGATCATCATCTGTGC. shVLDLR-2: Forward, 5′-CCGGGCTTGATTCTAAGTTGCACATCTCGAGATGTGCAACTTAGAATCAAGCTTTTTG; Reverse, 5′-AATTCAAAAAGCTTGATTCTAAGTTGCACATCTCGAGATGTGCAACTTAGAATCAAGC.

### Lentivirus preparation and stable cell line generation

Lentiviruses were packaged in HEK-293T cells using the second-generation packaging system plasmid psPAX2 (Addgene) and pMD2.G (Addgene). Transfection was performed with LipoD293 (SignaGen) following the manufacturer’s instructions. The lentivirus was harvested at both 24 h and 48 h after transfection and used for cell infection in the presence of 8 μg/ml polybrene. After infection for 48 h, the infected cells were selected with 2 μg/ml puromycin (Sigma–Aldrich) or 1 mg/ml G418 (Sigma–Aldrich).

### Recombinant protein purification

GST and GST-RAP fusion protein were expressed in bacteria and then purified. Briefly, recombined plasmid was expressed in *E. coli* Transetta (DE3) cells. At OD_600_ = 0.5, protein expression was induced with 1 mM IPTG (Solarbio) for 5 h at 25°C. Bacteria were lysed and sonicated in lysis buffer containing 50 mM Tris-HCl (pH 7.4), 300 mM NaCl, 0.5% Triton X-100, PMSF, DTT, lysozyme, and protease inhibitor cocktail (Sigma-Aldrich) at 4°C. After centrifugation at 12,000 *g* for 15 min at 4°C, the supernatant was used for the protein purification with GST resin (TransGen Biotech) according to the manufacturer’s instructions. Elution of recombinant protein was performed under mild, non-denaturing condition using reduced glutathione. Then, the fractions were collected and analyzed with SDS-PAGE followed by Coomassie brilliant blue staining.

Breast cancer cells were treated with 200 nM GST-RAP fusion protein for 72 h followed by immunofluorescent staining. Briefly, cells grown on slides were fixed with 4% paraformaldehyde. After blocking, slides were stained with anti-GST antibody (Proteintech, 10000-0-AP). Slides were then incubated with Alexa-488 Goat anti-Rabbit IgG (Invitrogen, A11008). Nuclei were stained with DAPI (Thermo Fisher Scientific).

### RNA extraction, cDNA synthesis, and quantitative PCR

Total RNA was extracted using Trizol reagent (Invitrogen) following the manufacturer’s instructions. The cDNA was generated using the EasyScript One-Step gDNA Removal and cDNA Synthesis SuperMix Kit (TransGen Biotech) according to the manufacturer’s instructions. The amount of cDNA used as the PCR template was equivalent to 60 ng of total RNA. The quantitative PCR reactions were performed by limiting cycle numbers from 28 to 30 dependent on the transcript examined. ACTB was used as an internal control for normalization. The PCR products were separated in a 2% agarose gel and detected by ethidium bromide staining. The primers were as follows: VLDLR: Forward, 5′-CAACCTGAATGATGCCCAAGA; Reverse, 5′-CTTTTGGGGGAACACTGACCT. ACTB: Forward, 5′-TTGCCGACAGGATGCAGAAGGA; Reverse, 5′-AGGTGGACAGCGAGGCCAGGAT. Each experiment was repeated three times.

### Western blot analysis

Cells were lysed on ice with RIPA buffer [150 mM NaCl, 0.5% sodium deoxycholate, 0.1% SDS, 1% NP40, and 50 mM Tris (pH 8.0)] supplemented with protease inhibitor cocktail (Sigma-Aldrich). Lysates were cleared by centrifuging at 12,000 rpm for 15 min. Next, the protein was quantified by the Coomassie brilliant blue dye method. After boiling with loading buffer for 5 min, equal amounts of cellular proteins were loaded to separate in SDS-PAGE and then transferred to a nitrocellulose membrane (Millipore) *via* submerged transfer. After blocking, the membranes were incubated overnight with primary antibodies at 4°C. Next, the membranes were incubated with HRP-conjugated secondary antibodies at room temperature for 1 h. The signals were visualized using an enhanced chemiluminescence kit (Advansta) according to the manufacturer’s instructions. The following antibodies were used: VLDLR (Proteintech, #19493-1), OCT4 (Abcam, #ab19857), SOX2 (Santa Cruz Biotechnology, #sc-365823), NANOG (Abcam, #ab109250), β-actin (Proteintech, #60008-1), Goat anti-Mouse IgG (HRP conjugated) (Thermo Fisher Scientific, #31430), and Goat anti-Rabbit IgG (HRP conjugated) (Thermo Fisher Scientific, #31460). Each experiment was repeated three times.

### CCK-8 assay

Cell proliferation was determined using CCK-8 kits (MedChemExpress) according to the manufacturer’s instructions. Briefly, cells were plated into 96-well microplates at 1 × 10^3^ cells/well density and the absorbance at 450 nm was measured every 24 h with a multimode plate reader (Perkin Elmer). Each experiment was repeated three times.

### Colony formation assay

Cells were plated into six-well plates at 500 cells/well density. Fresh growth medium was replaced every 3 days. After 7–10 days, colonies were fixed with 4% paraformaldehyde, stained with 0.5% crystal violet (Sigma-Aldrich) solution, washed, and dried. Images of stained plates were captured. Next, bound crystal violet was dissolved by 50% glacial acetic acid solution, and the absorbance was measured at 570 nm with a multimode plate reader (Perkin Elmer). Alternatively, the colony numbers were counted with ImageJ software. Each experiment was repeated three times.

### Sphere formation assay

Cells were plated into ultra-low attachment 96-well plates (Corning) at 500 cells/well density and cultured in DMEM/F12 (Gibco) supplemented with B27 (Invitrogen), 20 ng/ml epithelial growth factor (EGF, Sigma-Aldrich), 20 ng/ml basic fibroblast growth factor (bFGF, PeproTech), and 1% methylcellulose (Sigma-Aldrich). EV and VLDLR overexpressed cells were cultured for 10 days, while shNC and shVLDLR cells were cultured for 12 days. Each experiment was repeated three times. For serial sphere experiment, cells were plated into ultra-low attachment six-well plates (Corning) at 5,000 cells/well density and cultured without methylcellulose. One week later, single cells from digested spheres were used for the next generation of sphere formation. The spheres were photographed and counted under an inverted microscope (Olympus).

### Cell cycle analysis

Propidium iodide (PI, Sigma-Aldrich) staining was used to detect cell cycle distribution. Briefly, the cells were harvested, washed with cold PBS, and fixed in 70% ethanol overnight at 4°C. Cells were washed with PBS; resuspended in 500 µl of binding buffer containing PI, RNase (BD Pharmingen), and 0.01% Triton X-100; and then incubated for 15 min at room temperature in the dark. The cell cycle distribution was analyzed by CytoFLEX flow cytometry (Beckman Coulter). Single cells were gated and were used for cell cycle analysis. Each experiment was repeated three times.

### Cell apoptosis analysis

Cell apoptosis was determined by flow cytometry using an Annexin V-FITC/PI kit (Tianjin Sungene Biotech) according to the manufacturer’s instructions. Briefly, adherent and floating cells were collected, washed with PBS, and stained with Annexin V and PI in 1× binding buffer for 15 min at room temperature. Cells were further diluted with the buffer and analyzed using a CytoFLEX flow cytometry (Beckman Coulter). Cell population fractions in four quadrants were analyzed using FlowJo software. Cells were gated and were used for cell apoptosis analysis. The percentage of apoptotic cells (AV^+^/PI^‐^ and AV^+^/PI^+^) was calculated using the following formula: Apoptotic rate (%) = (number of apoptotic cells/total number of cells examined) × 100. Each experiment was repeated three times.

### Nile red staining

Nile red staining was used to assess the lipid content of cells. Nile red powder (Sigma-Aldrich) was dissolved in DMSO to make a 1 mg/ml stock solution, which was kept in the dark at −20°C. This stock solution was then diluted 1:100 in PBS before use. Staining was carried out on both fixed cells and unfixed cells. Cells were fixed in 4% paraformaldehyde and stained with Nile red solution for 15 min at room temperature. Nuclei were stained with DAPI. After washing with PBS, cells were photographed under fluorescent microscopy (Olympus). For FACS analysis, cells were harvested, washed with PBS, and incubated in 500 μl of Nile red solution for 15 min at room temperature. Stained cells were washed with PBS and analyzed using CytoFLEX flow cytometry (Beckman Coulter). Cells were gated and were used for data analysis. Mean fluorescence values were determined using FlowJo software. Each experiment was repeated three times.

### Tumor growth in xenografts

All animal studies were approved by the Institutional Animal Care and Use Committee of Dalian Medical University and were carried out in accordance with established institutional guidelines and approved protocols. MDA-MB-231-shNC and MDA-MB-231-shVLDLR cells (1 × 10^6^) in 100 μl PBS containing 50% Matrigel (BD Biosciences) were subcutaneously injected into the left and right dorsal flank of female BALB/c nude mice (4 weeks old), respectively. Drinking water containing 2 mg/ml doxycycline (Dox) and 3% sucrose was used 3 days before subcutaneous inoculation and replaced every 2 days. The body weight of mice and the two perpendicular diameters (length: a, width: b) of tumors were recorded every other day. Tumor volume (V) was calculated according to the standard formula (V = 0.5 × a × b^2^). At the end of experiment, xenografts were dissected from euthanized mice and then photographed.

### IHC and scoring

Following informed consent and in accordance with the guidelines of the Institutional Research Medical Ethics Committee, breast cancer specimens were obtained from the First Affiliated Hospital of Dalian Medical University.

The molecular subtypes of breast cancer were defined as follows: Luminal A: ER^+^ and/or PR^+^, HER2^-^, Ki-67 < 14%. Luminal B (HER2^-^): ER^+^ and/or PR^+^, HER2^-^, Ki−67 ≥ 14%. Luminal B (HER2^+^): ER^+^ and/or PR^+^, HER2^+^. HER2: ER^−^, PR^−^, HER2^+^. Triple-negative: ER^−^, PR^−^, HER2^−^.

Immunohistochemical (IHC) staining was performed to examine the expression of VLDLR in breast cancer tissues. Briefly, slides were deparaffinized, rehydrated, and subjected to antigen retrieval by heating the sample with citrate buffer (pH 6.0) in a microwave oven. Endogenous peroxidase activity was quenched by 3% H_2_O_2_. After blocking, sections were incubated with VLDLR primary antibody (1:50; Santa Cruz Biotechnology, #sc-18824) at 4°C overnight. Then, biotinylated secondary antibody, streptavidin-conjugated HRP, and DAB were applied for specific detection. Images were viewed using light microscopy (Olympus).

The IHC staining results were reviewed and scored independently by two pathologists who were blinded to specimens’ clinical information, based on both the intensity of staining and the percentage of positively stained tumor cells. The intensity of protein expression was shown as follows: 0 (no staining), 1 (weak staining), 2 (moderate staining), and 3 (strong staining). The histochemical score (H-score) was calculated as the product of the staining intensity and the percentage of positive cells.

### Protein preparation

Control (empty vector, EV) and VLDLR-I/II overexpressed cells were lysed with freshly prepared lysis buffer [100 mM NH_4_HCO_3_ buffer, pH 10.0, 6 M guanidine hydrochloride, and protease inhibitor cocktail (Sigma-Aldrich)] followed by ultrasonication at 4°C for 30 min. The lysate was incubated on ice for 15 min and then pelleted by centrifugation at 12,000 *g* for 15 min. A BCA protein assay kit (ThermoFisher, China) was used to determine the protein concentration of each sample. A total of 200 μg of protein from each lysate was reduced with 20 mM dithiothreitol (DTT) for 1 h at 55°C followed by protein alkylation with 40 mM iodoacetamide in the dark at room temperature for 30 min. After protein samples were desalted with ultrafiltration device (Vivacon 500, Sartorius) and digested overnight at 37°C with trypsin (Promega 1:50 w/w), the digested peptides were lyophilized and reconstituted in 50 mM HEPES (pH 8.0).

### Mass spectrometry

The lyophilized samples were dissolved in 0.1% trifluoroacetic acid (TFA) and quantified using the NanoDrop 2000 (Thermo Fisher Scientific). Approximately 1 μg of sample was run on the Q Exactive HF mass spectrometer coupled with the Ultimate 3000 RSLCnano system to collect data. The mass spectrometric data were processed by Xcalibur software (version 2.1.0, Thermo Fisher Scientific) and raw files were analyzed with MaxQuant software (version 1.5.3.30). All files were searched against the UniProt human reference database using the Andromeda search engine. Digestion mode was set to trypsin with a maximum of two missed cleavages. Carbamidomethylation of cysteine was set as a fixed modification. Variable modifications included N-terminal protein acetylation and methionine oxidation. The precursor mass tolerance and fragment mass tolerance were set at 10 ppm and 0.02 Da, respectively. The false discovery rate (FDR) was set at 0.01 to eliminate low-probability protein identifications.

### Bioinformatics analysis

VLDLR mRNA expression levels of monolayer and sphere-forming cells of breast cancer cell lines (MDA-MB-231-LM2, MCF7, and SUM159) were obtained from the GEO database (https://www.ncbi.nlm.nih.gov/geo/). Two original datasets were downloaded (GEO accession no. GSE98239 and GSE43657). VLDLR mRNA expression levels in normal breast tissues and breast cancer tissues were analyzed with the GENT2 database (http://gent2.appex.kr/gent2/). The prognostic significance of VLDLR was evaluated using the PROGgene V2 database (http://www.progtools.net/gene/), GENT2 database (http://gent2.appex.kr/gent2/), and GEPIA2 database (http://gepia2.cancer-pku.cn/#correlation). Gene correlation analysis was done using the GEPIA2 database (http://gepia2.cancer-pku.cn/#correlation).

### Statistics

Experiments were performed at least three times. Statistical analysis was performed using the GraphPad Prism statistical software (GraphPad Prism 8.0.2). All data were expressed as the mean ± SEM. The unpaired *t*-test was used to compare the difference between two groups. One-way ANOVA followed by Dunnett’s test was used for multiple group comparison. *p*-value < 0.05 was considered statistically significant (ns: no significance, *p < 0.05, **p < 0.01, ***p < 0.001).

## Results

### Expression of VLDLR is increased in breast cancer stem cell population

**Figure 1:**
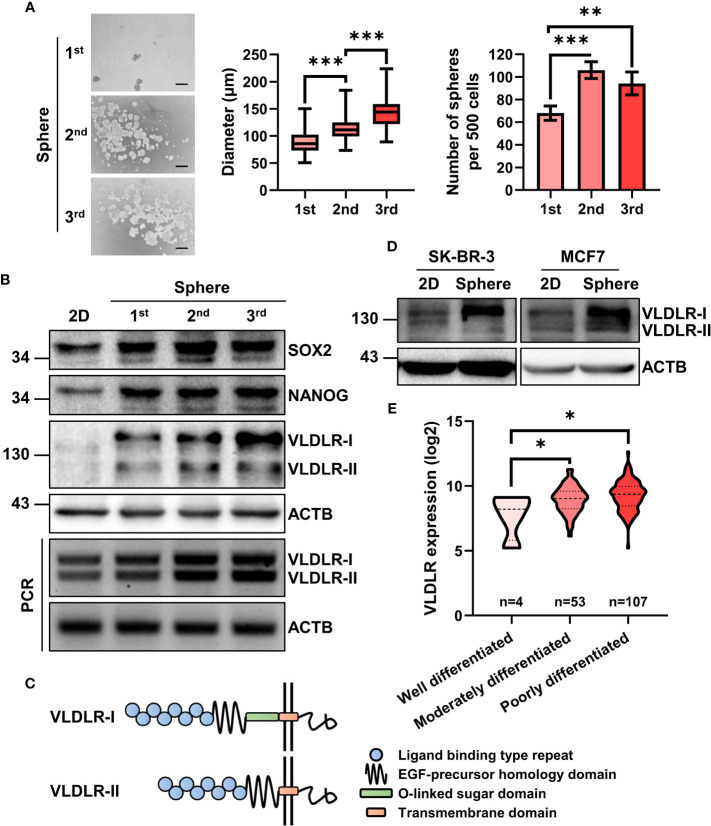
mRNA and protein expression of VLDLR are elevated in breast cancer stem cell population. **(A, B)** BCSCs in MDA-MB-231 cells were enriched by serial sphere formation assay. Representative image, size, and number of spheres were shown, respectively **(A)**. Scale bar: 200 μm. Data are presented as the mean ± SEM. Expression of indicated proteins and genes was analyzed by Western blot and reverse-transcriptase polymerase chain reaction (RT–PCR), respectively. ACTB was used as the internal control **(B)**. **(C)** Protein schematic of VLDLR variants (VLDLR-I and VLDLR-II). **(D)** BCSCs in SK-BR-3 and MCF7 cells was enriched by sphere formation assay for one generation. Expression of VLDLR-I and VLDLR-II was analyzed by Western blot. ACTB was used as the internal control. **(E)** The distribution of VLDLR mRNA expression in breast cancer tissues with different differentiation status was obtained from the GENT2 database. One-way ANOVA followed by Dunnett’s multiple comparisons test was used for statistical analysis. *p < 0.05, **p < 0.01, ***p < 0.001.

To enrich the BCSCs, three generations of sphere formation (18, 19) were performed on the triple-negative breast cancer cell line MDA-MB-231 (ER^−^PR^−^HER2^−^). The diameter and number of spheres were increased during serial passage (**Figure 1A**), indicating that BCSCs were successfully enriched. Consistently, the expression levels of SOX2 and NANOG, master stemness factors, were increased in spheres compared to monolayer cells (2D) (**Figure 1B**).

Proteomic analysis is a powerful high-throughput technique for understanding cellular protein expression. In this study, isobaric tags for relative and absolute quantification (iTRAQ) technology (20), a novel tool for the detection of protein expression, was used to assess proteomic changes of these three generations of spheres (unpublished data). From the proteomic data, we observed several cancer stem cell-related proteins, including STC1 (21), IL1B (22, 23), ICAM1 (24, 25), CEBPB (26), and HMGA1 (27), were upregulated during serial passage (**Supplementary Figure 1A** and **Supplementary Table 1**). Interestingly, VLDLR, a cell surface protein that has not been investigated in BCSCs, was gradually increased in both mRNA and protein level (**Supplementary Figures 1A, B** **)**. VLDLR has two variant forms because of alternative splicing (**Figure 1C**). We further found that both subtypes were gradually increased during serial sphere formation process (**Figure 1B**). We also enriched BCSCs from MCF7 (ER^+^PR^+^HER2^−^) and SK-BR-3 (ER^−^PR^+^HER2^+^) breast cancer cell lines by sphere formation assay for one generation and observed that VLDLR-I in SK-BR-3 and VLDLR-I and VLDLR-II in MCF7 were increased in sphere-forming cells compared to monolayer cells (2D) (**Figure 1D**). Furthermore, increased VLDLR mRNA expression levels were observed in sphere-forming cells from MCF7, SUM159 (ER^−^PR^−^HER2^−^) (28), and MDA-MB-231-LM2 cells (29, 30) (**Supplementary Figure 1C**), which exhibit enhanced lung metastasis capability, using the public GEO datasets (GSE43657 and GSE98239). Furthermore, VLDLR mRNA was significantly upregulated in moderately and poorly differentiated breast cancer tissues compared to well-differentiated compartments (**Figure 1E**). Taken together, these findings indicated that VLDLR was upregulated in BCSCs.

### VLDLR regulates BCSC stemness *in vitro* and *in vivo*

**Figure 2:**
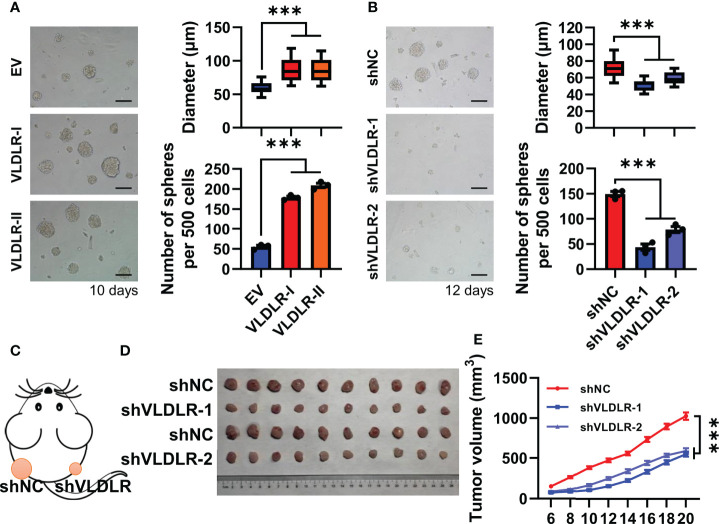
VLDLR regulates BCSC stemness *in vitro* and *in vivo*. **(A)** MDA-MB-231 cells overexpressing VLDLR-I/II were established, followed by sphere formation assay for 10 days. Representative image, size, and number of spheres were shown, respectively. EV: empty vector. **(B)** Sphere formation abilities of MDA-MB-231 cells were analyzed following VLDLR knockdown for 12 days. Representative image, size, and number of spheres were shown, respectively. Scale bar: 100 μm. **(C)** Control (shNC) and VLDLR knockdown (shVLDLR) MDA-MB-231 cells were injected into nude mice subcutaneously (1 × 10^6^ cells per injection site). Schematic diagram of xenograft experiment was shown. **(D)** Photograph of xenograft tumors dissected from mice (10 mice in each group). **(E)** Tumor volume was measured at the indicated time points. Data are presented as the mean ± SEM of three independent experiments. One-way ANOVA followed by Dunnett’s test was used for multiple group comparisons, ***p < 0.001.

To investigate the biological effect of different subtypes of VLDLR on breast cancer cell stemness, we stably overexpressed VLDLR-I/II in MDA-MB-231 cells (**Supplementary Figure 2A**) and found that the sphere formation capabilities were significantly enhanced by both VLDLR-I and VLDLR-II expression (**Figure 2A**). To prevent the adhesion of adjacent spheres, 1% methylcellulose was added in the sphere medium, resulting in the different morphology of spheres compared with those of serial sphere formation assay. We also developed two stable cell lines transduced with different VLDLR-specific shRNAs that can be induced by Dox. Western blot analysis showed that VLDLR expression was significantly decreased in monolayer MDA-MB-231 cells following Dox treatment for 5 days (**Supplementary Figure 2B**). These stable cell lines were subsequently applied in the sphere formation assay. Results demonstrated that VLDLR knockdown remarkably inhibited stem cell growth, and the numbers of spheres were reduced by more than 50% in VLDLR silenced cells compared to control cells (**Figure 2B**). These data suggested that VLDLR was essential for BCSC expansion.

To further determine the effects of VLDLR on tumor growth *in vivo*, we subcutaneously inoculated nude mice with MDA-MB-231 stable cells expressing inducible shNC or shVLDLRs into dorsal flank (**Figure 2C**). Dox dissolved in drinking water was employed to trigger the endogenous VLDLR knockdown of the xenograft throughout the experiment. Tumor volume was monitored from day 0 to day 20 after inoculation, and the results showed that the tumors derived from VLDLR silenced cells grew significantly slower than tumors derived from control cells (**Figures 2D, E**). Collectively, these results demonstrated that VLDLR silencing inhibited the tumor development *in vivo*.

### Transition to quiescence is induced by VLDLR silencing

To elucidate the potential mechanisms of VLDLR responsible for the BCSC characteristics, we detected the expression of three master stemness factors. Surprisingly, neither VLDLR-I nor VLDLR-II overexpression had detectable effects on the expression of OCT4, SOX2, and NANOG (**Supplementary Figure 2A**). Consistent with the results from overexpression experiments, no change was observed in the expression of these stemness-related proteins upon VLDLR silencing (**Supplementary Figure 2C**), indicating that the OCT4/SOX2/NANOG regulatory circuit was not affected. We then investigated whether VLDLR knockdown induced cell apoptosis in breast cancer cells. Cell apoptosis was analyzed by flow cytometry, and results showed that the percentages of Annexin V-positive cells were slightly increased but still lower than 3% (**Supplementary Figures 3A, B**). Given that the numbers of spheres were reduced by more than 50% upon VLDLR knockdown (**Figure 2B**), we proposed that the increased cell apoptosis was not enough to explain the inhibited cancer stem cell function in VLDLR silenced cells.

**Figure 3:**
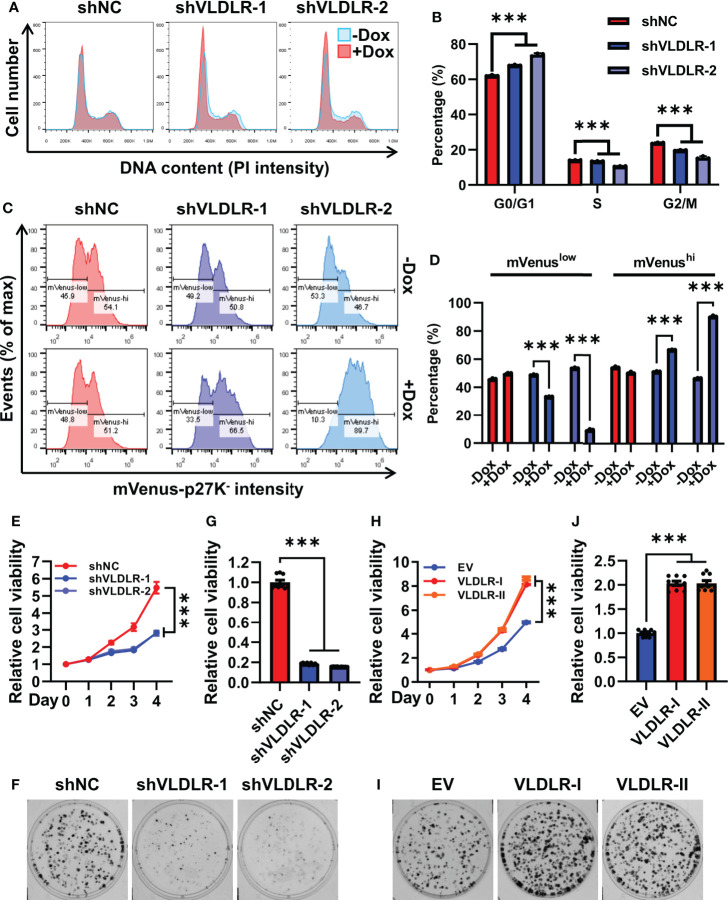
Transition to quiescence is induced by VLDLR silencing. **(A, B)** The cell cycle profiles of control (shNC) and VLDLR knockdown cells with or without Dox treatment were analyzed by flow cytometry **(A)**. Percentages of cells in G0/G1, S, and G2/M phases were shown **(B)**. **(C, D)** MDA-MB-231 cells were co-transfected with shRNA vector (shNC or shVLDLR) and the mVenus-p27K^-^ expression vector *via* lentivirus-mediated gene transfer. Puromycin and neomycin double-selected cells were treated with or without Dox for 5 days. The expression of mVenus-p27K^-^ was analyzed by flow cytometry **(C)**. The percentages of cells with low (mVenus^low^) or high (mVenus^hi^) mVenus-p27K^-^ intensity were shown **(D)**. **(E)** The proliferation abilities of control (shNC) and VLDLR knockdown cells were analyzed by CCK-8 assay. Relative cell viability was normalized to Day 0 of each cell line. **(F, G)** Colony formation abilities of control (shNC) and VLDLR knockdown cells were assessed. Cells were stained with crystal violet and representative photographs were shown **(F)**. For quantitative analysis, the bound crystal violet was completely dissolved with 50% glacial acetic acid and then the absorbance was measured at 570 nm. Relative cell viability was normalized to control (shNC) cells **(G)**. **(H)** The proliferation abilities of control (EV) and VLDLR-I/II overexpressed cells were analyzed by CCK-8 assay. Relative cell viability was normalized to Day 0 of each cell line. **(I, J)** Colony formation abilities of control (EV) and VLDLR-I/II overexpressed cells were assessed. Cells were stained with crystal violet and representative photographs were shown **(I)**. Relative cell viability was normalized to control (EV) cells **(J)**. EV: empty vector. Data are presented as the mean ± SEM of three independent experiments. The unpaired *t*-test was used to compare the difference between two groups. One-way ANOVA followed by Dunnett’s test was used for multiple group comparisons. ***p < 0.001.

Cell cycle progression is a precondition for the cancer cell proliferation. Upon VLDLR knockdown, we observed increased G0/G1 cell population and concomitant decreased S and G2/M population in MDA-MB-231 cells (**Figures 3A, B**), suggesting that VLDLR silencing induced G0/G1 phase cell cycle arrest. Cells with 2N DNA content were identified in either the G0 or G1 phase. The G1 phase is the first phase in which cells commit to enter the mitotic cycle and prepare to duplicate their DNA in the S phase. The G0 phase, however, is a reversible quiescent state in which cells remain viable but do not proliferate until they are stimulated into an active cell cycle. To distinguish between these two phases, we established a stable cell line expressing mVenus-p27K^-^ fusion protein, in which the mVenus fluorescent intensity can be used as an indicator of quiescence (31). Cell cycle phase distribution was analyzed by PI staining. As expected, cells with low fluorescent intensity were distributed in all cell cycle phases, while cells with high fluorescent intensity were only distributed in the G0/G1 phase (**Supplementary Figure 4A**). Furthermore, in this stable cell line, the percentage of cells with high mVenus fluorescent intensity was significantly increased following VLDLR knockdown (**Figures 3C, D**), indicating that VLDLR silencing promoted breast cancer cells to enter a quiescent state. Therefore, we concluded that the entry of cellular quiescence contributed to VLDLR knockdown-mediated breast cancer cell growth inhibition. Consistently, cell proliferation and colony formation capacity were remarkably blunted by VLDLR reduction (**Figures 3E–G**), which can be rescued by VLDLR restoration (**Supplementary Figures 4B, C**). In contrast, VLDLR-I and VLDLR-II overexpression markedly enhanced the proliferation and colony growth of cells (**Figures 3H–J**). Taken together, VLDLR is essential for breast cancer cell proliferation.

### VLDLR promotes breast cancer cell proliferation in a ligand- and lipid-independent manner

VLDLR has been reported to play essential roles in lipid metabolism and signal transduction by binding to numerous ligands. We then investigated whether these functions are involved in VLDLR-mediated promotion of breast cancer cell growth.

**Figure 4:**
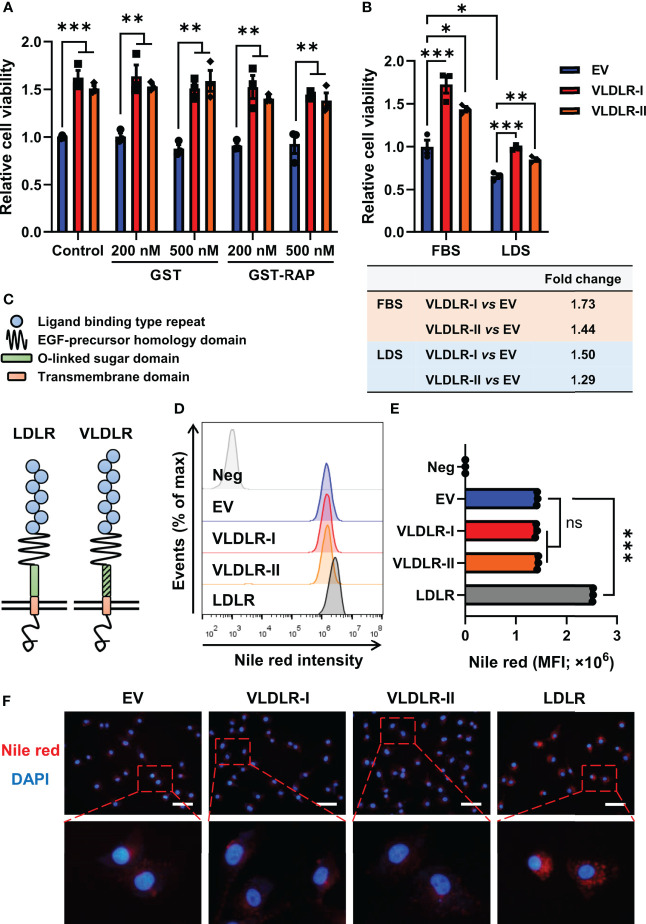
VLDLR promotes breast cancer cell proliferation in a ligand- and lipid-independent manner. **(A)** Control (EV) and VLDLR-I/II overexpressed cells were treated for 72 h with the indicated concentrations of GST or GST-RAP fusion protein. Cell viability was measured by CCK-8 assay. Relative cell viability was normalized to control (EV) cells without treatment. **(B)** Control (EV) and VLDLR-I/II overexpressed cells were cultured for 72 h with 10% FBS or LDS. Cell viability was measured by CCK-8 assay. Relative cell viability was normalized to control (EV) cells cultured with 10% FBS. The fold change of cell viability of VLDLR-I/II overexpressed cells compared to control (EV) cells under each culture condition was calculated and shown on the bottom. **(C)** Schematic of LDLR and VLDLR protein structure. **(D, E)** VLDLR-I/II and LDLR overexpressed cells were stained with Nile red, analyzed by flow cytometry, and compared with control (EV) cells. Negative control was performed by incubating the cells without Nile red. MFI, mean fluorescence intensity. **(F)** Cells derived from **(D)** were double-stained with Nile red and DAPI. Representative images were shown. Scale bar: 50 μm. EV: empty vector. Data are presented as the mean ± SEM of three independent experiments. The unpaired *t*-test was used to compare the difference between two groups. One-way ANOVA followed by Dunnett’s test was used for multiple group comparisons. ns: no significance, *p < 0.05, **p < 0.01, ***p < 0.001.

The 39-kDa RAP, a molecular chaperone, can bind with high affinity to VLDLR, thereby blocking the binding of other ligands to the receptor (6). In this study, GST and the GST-RAP fusion protein were purified (**Supplementary Figure 5A**) and added to the cell culture medium. No significant change in proliferation of MDA-MB-231 cells was found after GST-RAP treatment (**Figure 4A** and **Supplementary Figure 5B**), indicating that ligand-independent activities may be involved in VLDLR-mediated promotion of breast cancer cell growth.

VLDLR can promote intracellular lipid accumulation through VLDL uptake. To exclude the effect of lipid in the medium, we cultured cells with medium containing 10% FBS or 10% LDS. Interestingly, VLDLR-I and VLDLR-II overexpression in MDA-MB-231 cells significantly promoted cell growth even under lipid depleted condition (**Figure 4B**). Even though VLDLR promoted cell growth at a lower level under lipid-depleted conditions, our results suggested that the lipid-independent function of VLDLR was involved in cancer cell proliferation. Furthermore, cellular lipid was stained with Nile red, a fluorescent neutral lipid stain, and analyzed *via* flow cytometry. VLDLR exhibits structural similarity to those of the LDLR, except VLDLR has an extra repeat of the cysteine-rich ligand-binding domain (**Figure 4C**). LDLR, an important cell surface receptor, mediates lipid uptake (32), and its role in the progression of familial hypercholesterolemia has been widely studied (33). Therefore, we used LDLR overexpressed cells as a positive control in this experiment. Results showed that the mean fluorescent intensity (MFI) of Nile red in VLDLR-I or VLDLR-II overexpressed cells was not increased compared to control cells (**Figures 4D, E**). Moreover, fixed cells were stained with Nile red and DAPI, and the results were consistent with flow cytometry (**Figure 4F**). In contrast, LDLR overexpression significantly stimulated lipid accumulation in breast cancer cells (**Figures 4D–F**). As expected, VLDLR knockdown did not inhibit lipid accumulation in breast cancer cells (**Supplementary Figures 6A, B**). These results demonstrated that the promotion of cell growth is independent of the lipid metabolism activity of VLDLR in breast cancer cells.

### VLDLR expression correlates with elevated TCA cycle and ribosome biogenesis

**Figure 5:**
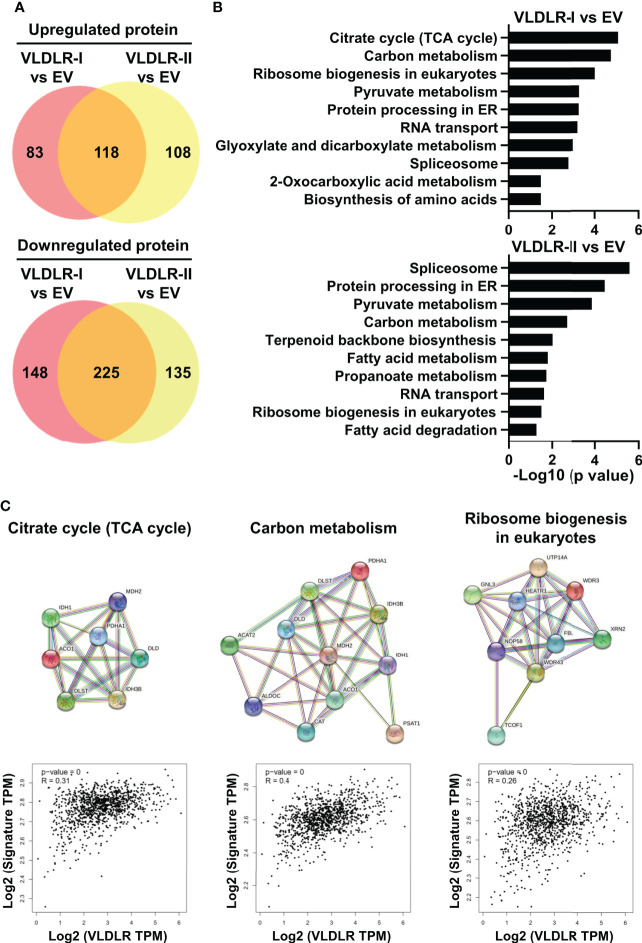
VLDLR expression correlates with elevated TCA cycle and ribosome biogenesis. **(A)** The whole cell lysates of control (EV) and VLDLR-I/II overexpressed cells were used for quantitative mass spectrometry. Venn diagrams of up- and downregulated proteins in VLDLR-I/II overexpressed cells compared to control (EV) cells (>1.2-fold) were shown. EV: empty vector. **(B)** Biological process enrichment analysis of upregulated proteins in VLDLR-I/II overexpressed cells. **(C)** Interactions of upregulated proteins involved in TCA cycle, carbon metabolism, and ribosome biogenesis in VLDLR-I overexpressed cells were analyzed with string database (upper panel). The mRNA expression correlation between VLDLR and these gene signatures was analyzed with GEPIA2 database (lower panel).

To explore the possible mechanism by which VLDLR-I/II promotes cancer cell proliferation, we performed proteomic analysis in control and VLDLR-I/II overexpressed MDA-MB-231 cells. Results demonstrated that more than half of upregulated and downregulated proteins were shared in VLDLR-I and VLDLR-II overexpressed cells compared to control cells (**Figure 5A**, **Supplementary Table 2**), indicating that VLDLR-I and VLDLR-II have similar regulations of protein expression in cancer cells. Biological process enrichment analysis indicated that upregulated proteins upon VLDLR-I/II overexpression were associated with citrate cycle (TCA cycle), carbon metabolism, and ribosome biogenesis (**Figures 5B, C**). Furthermore, in breast cancer tissues, the expression of VLDLR mRNA exhibits positive correlations with gene signatures constituted by genes involved in these pathways (**Figure 5C**, lower panel), suggesting that VLDLR expression correlated with elevated energy production and ribosome biogenesis.

### Elevated VLDLR expression predicts poor prognosis of breast cancer

**Figure 6:**
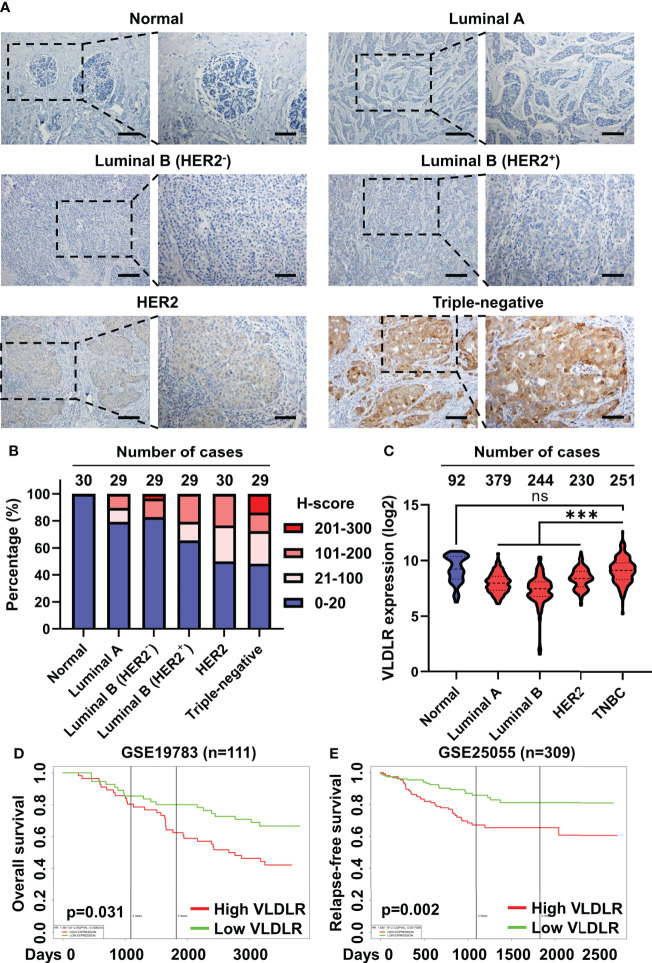
Elevated VLDLR expression predicts poor prognosis of breast cancer. **(A)** Representative images of immunohistochemical staining for VLDLR in normal breast tissues and various breast cancer subtypes. Scale bar: 100 μm in lower-magnification images, 50 μm in the enlarged images. **(B)** The distribution of H-scores across normal breast tissues and various breast cancer subtypes. **(C)** The distribution of VLDLR mRNA expression across normal breast tissues and various breast cancer subtypes was obtained from the GENT2 database. One-way ANOVA followed by Dunnett’s multiple comparisons test was used for statistical analysis. ns: no significance, ***p < 0.001. **(D)** Overall survival of 111 breast cancer patients with high or low expression of VLDLR was analyzed using PROGgeneV2. Dataset from cohort GSE19783. **(E)** Relapse-free survival of 309 breast cancer patients with high or low expression of VLDLR was analyzed using PROGgeneV2. Dataset from cohort GSE25055.

To explore the role of VLDLR in breast cancer, we examined the expression levels of VLDLR in 146 cases of breast cancer tissues and 30 control subjects by immunohistochemistry. Although low/negative expression of VLDLR (H-score ≤ 20) was observed in more than half of the breast cancer cases, positive expression of VLDLR (H-score > 20) occurred more frequently in breast cancer tissues than in tumor adjacent normal breast tissues (**Figures 6A, B**). Moreover, strong staining (H-score > 200) was observed in four cases (13.8%) of the triple-negative subtype (**Figure 6B**), which has the highest degree of malignancy and the worst prognosis among all subtypes (34). Furthermore, by analyzing VLDLR expression in the GENT2 database (35), we observed that the VLDLR mRNA expression levels were significantly increased in triple-negative breast cancer (TNBC) compared to other subtypes (**Figure 6C**). Consistently, VLDLR mRNA expression levels were higher in ER- and PR-negative tissues than positive compartments (**Supplementary Figure 7A**, left and middle panel). The correlation between VLDLR and HER2 was not observed (**Supplementary Figure 7A**, right panel).

To further validate the important role of VLDLR in the outcome of breast cancer patients, we stratified the breast cancer patients based on the VLDLR expression level using a publicly available database (35–37) and found that elevated VLDLR expression was significantly associated with worse overall survival (**Figure 6D** and **Supplementary Figure 7B**), as well as relapse-free survival (**Figure 6E**). However, this correlation was not observed in the GEPIA2 database (**Supplementary Figure 7C**). We further analyzed overall survival of breast cancer patients in separated subtypes and found that high VLDLR expression significantly correlated with poor prognosis in TNBC patients in contrast to other subtypes (**Supplementary Figure 7D**). Taken together, these results suggested that VLDLR may act as an important protein to promote cancer progression in human breast tumors, especially in TNBC.

## Discussion

Cancer stem cells (CSCs), defined by their ability to self-renew and to regenerate a tumor upon transplantation, play pivotal roles in tumor initiation and progression (38). CD44^+^/CD24^-^ (39) and aldehyde dehydrogenase 1 (ALDH1) (40) are the most widely used markers to identify BCSCs. Currently, accumulating studies have identified more BCSC markers, such as PROCR (41), ANTXR1 (42), and GPNMB (43). However, antitumor strategies that specifically target BCSCs are still limited. Thus, identification of therapeutic targets of BCSCs is urgently needed. In the present study, serial sphere formation assay, a widely used *in vitro* technique for assessing self-renewal capacity (18, 19), was performed to enrich BCSCs. During this process, both mRNA and protein of VLDLR were upregulated (**Figure 1B** and **Supplementary Figures 1A, B**). Some studies found that VLDLR overexpression promotes cell proliferation and migration (12, 13, 16), but the role of VLDLR in BCSCs has not been reported. We demonstrated that targeting the expression of VLDLR in breast cancer cells dramatically decreased the sphere formation capacity *in vitro* and tumor growth *in vivo* (**Figures 2B–E**), suggesting that VLDLR plays a critical role in the BCSC phenotype. In our *in vivo* experiments, the breast cancer cells were subcutaneously inoculated into dorsal flank of mice. Given that cancer is influenced by the surrounding microenvironment, a mammary fat pad injection model may improve the relevant findings in a manner that better mimics human pathology. In addition, we did not detect any apparent metastatic nodule in these mice. The metastasis-promoting function needs further investigation with the tail vein injection metastasis model.

VLDLR exists in two variants, VLDLR-I encoded by all 19 exons and VLDLR-II lacking the O-linked sugar domain encoded by the 16th exon (4). Although VLDLR-II has been reported as the predominant form in breast cancer tissues (14), the specific functions of VLDLR-I and VLDLR-II in breast cancer are poorly understood. In gastrointestinal adenocarcinoma, VLDLR-II is predominantly expressed in poorly or moderately differentiated tissues, whereas VLDLR-I is mainly detected in well-differentiated tissues (17). Moreover, cell differentiation induced by all-trans retinoic acid (ATRA) was accompanied by attenuated cell proliferation and significantly decreased VLDLR-II expression in a gastric adenocarcinoma cell line. In contrast, phorbol-12-myristate-13-acetate (PMA) promoted cell proliferation and enhanced VLDLR-II expression (16). These results suggest that VLDLR-II may be involved in cancer cell differentiation. In addition, contradictory functions of VLDLR-I and VLDLR-II were observed in the gastric adenocarcinoma cell line SGC7901. Overexpressed VLDLR-II induced cell proliferation and the activation of β-catenin/T-cell factor (TCF) signaling, whereas VLDLR-I overexpression had the opposite effect (16). In the present study, we found that both VLDLR-I and VLDLR-II were upregulated in enriched BCSCs (**Figure 1B**), which is in line with their sphere-promoting functions (**Figure 2A**). We proposed that the different functions of VLDLR-I may be context-dependent.

Cancer cell stemness is regulated by a strong transcriptional circuit constituted by OCT4, SOX2, and NANOG (38). Surprisingly, neither VLDLR-I/II overexpression nor knockdown showed detectable effect on the expression of these three proteins (**Supplementary Figures 2A, C**). We then thought that VLDLR may directly regulate the behavior of BCSCs. Cell cycle progression is a precondition for cancer cell proliferation. We then found that VLDLR inhibition resulted in G0/G1 arrest (**Figures 3A, B**). Cancer cell quiescence is defined as a reversible state of growth arrest in which cells enter the G0 phase of the cell cycle. Cancer cells can escape chemotherapy by entering a quiescent state (44). However, cancer cells in active cell cycle can produce large amounts of descendants to promote cancer progression. We used mVenus-p27K^-^ (31), a fluorescent indicator of cell quiescence, to investigate the function of VLDLR in this process. Results indicated that VLDLR inhibition induced quiescence in breast cancer cells (**Figures 3C, D**). Consistently, our data revealed that silencing endogenous VLDLR inhibited cell proliferation and colony formation ability in breast cancer cells (**Figures 3E–G**), whereas VLDLR-I and VLDLR-II overexpression caused the opposite effects (**Figures 3H–J**). Given that conventional therapies, including chemotherapy, usually target highly proliferative cells, VLDLR silencing-induced cell quiescence may contribute to the survival of the BCSC population. However, we presented that cancer cell proliferation was highly dependent on VLDLR expression, suggesting that cancer recurrence is difficult to occur without VLDLR expression. It should be noted that the above functional analyses were conducted with 2D cultured cells, which was different from their 3D cultured counterpart. Whether these functions were affected by VLDLR under 3D culture conditions needs further investigation.

VLDLR has been considered as a multifunctional receptor due to its ability to bind numerous ligands. The uPA-PAI-1 complex promoted breast cancer cell growth. However, this activity was not observed with the uPA-PAI-1(R76E) complex, which binds to the VLDLR with greatly decreased affinity (12). During brain development, Reelin binds to VLDLR and ApoER2, inducing tyrosine phosphorylation of Disabled-1 (Dab1), which is important for neuronal positioning (10). Previous studies have revealed that Reelin is downregulated in pancreatic cancer (45), colorectal cancer (46), and neuroblastoma (47). Moreover, silenced Reelin promotes cancer cell motility and colony-formation ability in pancreatic cancer cells (45). RAP, a molecular chaperone, can block the ligand binding activity of VLDLR (6). We treated cancer cells with GST-RAP fusion protein and found that cell proliferation promoted by VLDLR-I and VLDLR-II overexpression was not affected (**Figure 4A**), suggesting that the ligand binding activity was not involved in the cancer-promoting function of VLDLR.

Proliferating cancer cells require a large amount of lipids for energy production and plasma membrane synthesis (48). VLDLR expression facilitates lipid accumulation in adipose tissue (49). VLDL, a ligand of VLDLR, promotes breast cancer cell proliferation, metastasis, and angiogenesis (50). A previous study showed that expression of VLDLR was significantly increased in human clear-cell renal cell carcinoma (RCC) biopsies, which are characterized histologically by accumulation of cholesterol. Moreover, VLDLR knockdown reduced the lipid accumulation. These studies strongly suggested the important role of VLDLR in lipid accumulation (51). In the present study, breast cancer cells were cultured in DMEM supplemented with 10% LDS instead of FBS. Results showed that, even though lipid-deficient environment inhibited cell proliferation in all groups, overexpression of VLDLR-I or VLDLR-II promoted cell proliferation under this condition (**Figure 4B**). LDLR, with five structural domains very similar to those in VLDLR, plays a crucial role in lipoprotein metabolism (32). Familial hypercholesterolemia is a hereditary disease primarily due to mutations in the LDLR gene and characterized by strikingly elevated plasma levels of low-density lipoprotein cholesterol (LDL-C) (33). However, VLDLR knockout mice exhibited no lipoprotein abnormalities (52). In this study, neither VLDLR-I nor VLDLR-II overexpression significantly increased lipid content in breast cancer cells, while LDLR overexpression dramatically enhanced lipid accumulation (**Figures 4D–F**). Collectively, lipid-independent function of VLDLR was involved in cancer cell proliferation.

We found that more than half of the upregulated and downregulated proteins were shared in VLDLR-I and VLDLR-II overexpressed cells compared to control cells by the proteomic data (**Figure 5A**). The TCA cycle and ribosome biogenesis-related proteins were significantly enriched in upregulated proteins (**Figures 5B, C**). Furthermore, the expression of VLDLR exhibits significant correlations with gene signatures constituted by genes involved in these pathways (**Figure 5C**, lower panel), suggesting that VLDLR expression correlated with elevated energy production and ribosome biogenesis. However, whether these pathways are regulated by VLDLR or only the accompanying phenotypes of rapid cell growth requires further investigation.

Overexpression of VLDLR has been reported in numerous types of cancer, including breast cancer, in which VLDLR-II was predominantly expressed (14, 15, 53, 54). In the current study, we observed high expression of VLDLR in part of the breast cancer tissues, particularly in triple-negative breast cancer tissues, and reduced expression of VLDLR in normal breast tissues (**Figures 6A, B**). Analysis with the GENT2 database revealed that VLDLR mRNA expression was significantly higher in triple-negative breast cancer than in other subtypes (**Figure 6C**). Our data showed that the normal breast tissues and TNBC samples presented comparable VLDLR mRNA expression (**Figure 6C**) and different protein expression (**Figures 6A, B**). We thought of three possible reasons. First, the mRNA levels of VLDLR were examined with tumor tissues, which comprised cancer cells and non-cancer cells within the tumor microenvironment, possibly making the evaluation of VLDLR mRNA inaccurate. Second, as shown in **Figures 6A, B**, only part of breast cancer tissues presented high VLDLR expression. Third, different posttranscriptional regulation of VLDLR expression may exist between normal and malignant breast tissues, which needs further investigation. Furthermore, high expression of VLDLR was associated with poor overall survival and relapse-free survival in breast cancer patients (**Figures 6D, E** and **Supplementary Figures 7B, D**), suggesting that VLDLR could be considered as a potential therapeutic target for breast cancer therapy.

In summary, our study demonstrated that both VLDLR-I and VLDLR-II were upregulated in BCSCs. VLDLR silencing induced transition to quiescence of breast cancer cells in a ligand- and lipid-independent manner. VLDLR was upregulated in breast cancer tissues, especially TNBC tissues, and high expression of VLDLR predicts poor breast cancer prognosis. All these provide strong evidence for proposing VLDLR as a potential target for breast cancer treatment.

## Data availability statement

The original contributions presented in the study are included in the article/**Supplementary Material**. Further inquiries can be directed to the corresponding authors.

## Ethics statement

The studies involving human participants were reviewed and approved by the Ethics Committee of the First Affiliated Hospital of Dalian Medical University. The patients/participants provided their written informed consent to participate in this study. The animal study was reviewed and approved by the Institutional Animal Care and Use Committee of Dalian Medical University.

## Author contributions

QL, XC and MYY performed study concept and design. MYY, YZ and ZH performed the experiments, with participation from CW, WF, TG, ZL, LF, SLi and CG. SLv, HQ and MLY executed proteomic analysis. QL, XC, MYY and YZ wrote the manuscript. QL and XC led the project and oversaw preparation of the manuscript. All authors contributed to the article and approved the submitted version.

## References

[1] Siegel RL Miller KD Fuchs HE Jemal A. Cancer statistics, 2021. CA Cancer J Clin (2021) 71(1):7–33. doi: 10.3322/caac.21654 33433946

[2] Zeng X Liu C Yao J Wan H Wan G Li Y. Breast cancer stem cells, heterogeneity, targeting therapies and therapeutic implications. Pharmacol Res (2021) 163:105320. doi: 10.1016/j.phrs.2020.105320 33271295

[3] Go GW Mani A. Low-density lipoprotein receptor (LDLR) family orchestrates cholesterol homeostasis. Yale J Biol Med (2012) 85(1):19–28. 22461740 PMC3313535

[4] Iijima H Miyazawa M Sakai J Magoori K Ito MR Suzuki H. Expression and characterization of a very low density lipoprotein receptor variant lacking the O-linked sugar region generated by alternative splicing. J Biochem (1998) 124(4):747–55. doi: 10.1093/oxfordjournals.jbchem.a022175 9756619

[5] Takahashi S Suzuki J Kohno M Oida K Tamai T Miyabo S. Enhancement of the binding of triglyceride-rich lipoproteins to the very low density lipoprotein receptor by apolipoprotein e and lipoprotein lipase. J Biol Chem (1995) 270(26):15747–54. doi: 10.1074/jbc.270.26.15747 7797576

[6] Battey FD Gafvels ME FitzGerald DJ Argraves WS Chappell DA Strauss JF 3rd. The 39-kDa receptor-associated protein regulates ligand binding by the very low density lipoprotein receptor. J Biol Chem (1994) 269(37):23268–73. doi: 10.1016/S0021-9258(17)31648-4 8083232

[7] Mikhailenko I Krylov D Argraves KM Roberts DD Liau G Strickland DK. Cellular internalization and degradation of thrombospondin-1 is mediated by the amino-terminal heparin binding domain (HBD). high affinity interaction of dimeric HBD with the low density lipoprotein receptor-related protein. J Biol Chem (1997) 272(10):6784–91. doi: 10.1074/jbc.272.10.6784 9045712

[8] Argraves KM Battey FD MacCalman CD McCrae KR Gafvels M Kozarsky KF. The very low density lipoprotein receptor mediates the cellular catabolism of lipoprotein lipase and urokinase-plasminogen activator inhibitor type I complexes. J Biol Chem (1995) 270(44):26550–7. doi: 10.1074/jbc.270.44.26550 7592875

[9] Kasza A Petersen HH Heegaard CW Oka K Christensen A Dubin A. Specificity of serine proteinase/serpin complex binding to very-low-density lipoprotein receptor and alpha2-macroglobulin receptor/low-density-lipoprotein-receptor-related protein. Eur J Biochem (1997) 248(2):270–81. doi: 10.1111/j.1432-1033.1997.00270.x 9346278

[10] Lee GH D'Arcangelo G. New insights into reelin-mediated signaling pathways. Front Cell Neurosci (2016) 10:122. doi: 10.3389/fncel.2016.00122 27242434 PMC4860420

[11] Webb DJ Nguyen DH Sankovic M Gonias SL. The very low density lipoprotein receptor regulates urokinase receptor catabolism and breast cancer cell motility *in vitro* . J Biol Chem (1999) 274(11):7412–20. doi: 10.1074/jbc.274.11.7412 10066806

[12] Webb DJ Thomas KS Gonias SL. Plasminogen activator inhibitor 1 functions as a urokinase response modifier at the level of cell signaling and thereby promotes MCF-7 cell growth. J Cell Biol (2001) 152(4):741–52. doi: 10.1083/jcb.152.4.741 PMC2195772 11266465

[13] Zhou H Guo W Zhao Y Wang Y Zha R Ding J. MicroRNA-135a acts as a putative tumor suppressor by directly targeting very low density lipoprotein receptor in human gallbladder cancer. Cancer Sci (2014) 105(8):956–65. doi: 10.1111/cas.12463 PMC4317855 24903309

[14] Martensen PM Oka K Christensen L Rettenberger PM Petersen HH Christensen A. Breast carcinoma epithelial cells express a very low-density lipoprotein receptor variant lacking the O-linked glycosylation domain encoded by exon 16, but with full binding activity for serine proteinase/serpin complexes and Mr-40,000 receptor-associated protein. Eur J Biochem (1997) 248(2):583–91. doi: 10.1111/j.1432-1033.1997.00583.x 9346319

[15] He L Lu Y Wang P Zhang J Yin C Qu S. Up-regulated expression of type II very low density lipoprotein receptor correlates with cancer metastasis and has a potential link to beta-catenin in different cancers. BMC Canc (2010) 10:601. doi: 10.1186/1471-2407-10-601 PMC2988033 21047397

[16] Yang P Liu Z Wang H Tian J Li Y Zong Y. Enhanced activity of very low density lipoprotein receptor II promotes SGC7901 cell proliferation and migration. Life Sci (2009) 84(13-14):402–8. doi: 10.1016/j.lfs.2008.12.020 19167408

[17] Chen T Wu F Chen FM Tian J Qu S. Variations of very low-density lipoprotein receptor subtype expression in gastrointestinal adenocarcinoma cells with various differentiations. World J Gastroenterol (2005) 11(18):2817–21. doi: 10.3748/wjg.v11.i18.2817 PMC4305924 15884130

[18] Grimshaw MJ Cooper L Papazisis K Coleman JA Bohnenkamp HR Chiapero-Stanke L. Mammosphere culture of metastatic breast cancer cells enriches for tumorigenic breast cancer cells. Breast Cancer Res (2008) 10(3):R52. doi: 10.1186/bcr2106 18541018 PMC2481500

[19] Tong M Deng Z Yang M Xu C Zhang X Zhang Q. Transcriptomic but not genomic variability confers phenotype of breast cancer stem cells. Cancer Commun (Lond) (2018) 38(1):56. doi: 10.1186/s40880-018-0326-8 30231942 PMC6146522

[20] Wiese S Reidegeld KA Meyer HE Warscheid B. Protein labeling by iTRAQ: a new tool for quantitative mass spectrometry in proteome research. Proteomics. (2007) 7(3):340–50. doi: 10.1002/pmic.200600422 17177251

[21] Peng F Xu J Cui B Liang Q Zeng S He B. Oncogenic AURKA-enhanced N(6)-methyladenosine modification increases DROSHA mRNA stability to transactivate STC1 in breast cancer stem-like cells. Cell Res (2021) 31(3):345–61. doi: 10.1038/s41422-020-00397-2 PMC8027457 32859993

[22] Oh K Lee OY Park Y Seo MW Lee DS. IL-1beta induces IL-6 production and increases invasiveness and estrogen-independent growth in a TG2-dependent manner in human breast cancer cells. BMC Canc (2016) 16(1):724. doi: 10.1186/s12885-016-2746-7 PMC5017052 27609180

[23] Yu A Wang Y Bian Y Chen L Guo J Shen W. IL-1beta promotes the nuclear translocaiton of S100A4 protein in gastric cancer cells MGC803 and the cell's stem-like properties through PI3K pathway. J Cell Biochem (2018) 119(10):8163–73. doi: 10.1002/jcb.26813 29932233

[24] Liu S Li N Yu X Xiao X Cheng K Hu J. Expression of intercellular adhesion molecule 1 by hepatocellular carcinoma stem cells and circulating tumor cells. Gastroenterology. (2013) 144(5):1031–41.e10. doi: 10.1053/j.gastro.2013.01.046 23376424

[25] Tsai ST Wang PJ Liou NJ Lin PS Chen CH Chang WC. ICAM1 is a potential cancer stem cell marker of esophageal squamous cell carcinoma. PloS One (2015) 10(11):e0142834. doi: 10.1371/journal.pone.0142834 26571024 PMC4646358

[26] LaMarca HL Visbal AP Creighton CJ Liu H Zhang Y Behbod F. CCAAT/enhancer binding protein beta regulates stem cell activity and specifies luminal cell fate in the mammary gland. Stem Cells (2010) 28(3):535–44. doi: 10.1002/stem.297 PMC3006225 20054865

[27] Pegoraro S Ros G Piazza S Sommaggio R Ciani Y Rosato A. HMGA1 promotes metastatic processes in basal-like breast cancer regulating EMT and stemness. Oncotarget. (2013) 4(8):1293–308. doi: 10.18632/oncotarget.1136 PMC3787158 23945276

[28] Wang H Zhang Y Du Y. Ovarian and breast cancer spheres are similar in transcriptomic features and sensitive to fenretinide. BioMed Res Int (2013) 2013:510905. doi: 10.1155/2013/510905 24222909 PMC3816214

[29] Minn AJ Gupta GP Siegel PM Bos PD Shu W Giri DD. Genes that mediate breast cancer metastasis to lung. Nature. (2005) 436(7050):518–24. doi: 10.1038/nature03799 PMC1283098 16049480

[30] Insua-Rodriguez J Pein M Hongu T Meier J Descot A Lowy CM. Stress signaling in breast cancer cells induces matrix components that promote chemoresistant metastasis. EMBO Mol Med (2018) 10(10). doi: 10.15252/emmm.201809003 PMC6180299 30190333

[31] Oki T Nishimura K Kitaura J Togami K Maehara A Izawa K. A novel cell-cycle-indicator, mVenus-p27K-, identifies quiescent cells and visualizes G0-G1 transition. Sci Rep (2014) 4:4012. doi: 10.1038/srep04012 24500246 PMC3915272

[32] Zhao H Li Y He L Pu W Yu W Li Y. *In vivo* AAV-CRISPR/Cas9-Mediated gene editing ameliorates atherosclerosis in familial hypercholesterolemia. Circulation. (2020) 141(1):67–79. doi: 10.1161/CIRCULATIONAHA.119.042476 31779484

[33] Varghese MJ. Familial hypercholesterolemia: A review. Ann Pediatr Cardiol (2014) 7(2):107–17. doi: 10.4103/0974-2069.132478 PMC4070199 24987256

[34] Yin L Duan JJ Bian XW Yu SC. Triple-negative breast cancer molecular subtyping and treatment progress. Breast Cancer Res (2020) 22(1):61. doi: 10.1186/s13058-020-01296-5 32517735 PMC7285581

[35] Park SJ Yoon BH Kim SK Kim SY. GENT2: an updated gene expression database for normal and tumor tissues. BMC Med Genomics (2019) 12(Suppl 5):101. doi: 10.1186/s12920-019-0514-7 31296229 PMC6624177

[36] Goswami CP Nakshatri H. PROGgeneV2: enhancements on the existing database. BMC Canc (2014) 14:970. doi: 10.1186/1471-2407-14-970 PMC4300843 25518851

[37] Tang Z Kang B Li C Chen T Zhang Z. GEPIA2: an enhanced web server for large-scale expression profiling and interactive analysis. Nucleic Acids Res (2019) 47(W1):W556–W60. doi: 10.1093/nar/gkz430 PMC6602440 31114875

[38] Huang T Song X Xu D Tiek D Goenka A Wu B. Stem cell programs in cancer initiation, progression, and therapy resistance. Theranostics (2020) 10(19):8721–43. doi: 10.7150/thno.41648 PMC7392012 32754274

[39] Al-Hajj M Wicha MS Benito-Hernandez A Morrison SJ Clarke MF. Prospective identification of tumorigenic breast cancer cells. Proc Natl Acad Sci U S A (2003) 100(7):3983–8. doi: 10.1073/pnas.0530291100 PMC153034 12629218

[40] Ginestier C Hur MH Charafe-Jauffret E Monville F Dutcher J Brown M. ALDH1 is a marker of normal and malignant human mammary stem cells and a predictor of poor clinical outcome. Cell Stem Cell (2007) 1(5):555–67. doi: 10.1016/j.stem.2007.08.014 PMC2423808 18371393

[41] Wang D Hu X Liu C Jia Y Bai Y Cai C. Protein c receptor is a therapeutic stem cell target in a distinct group of breast cancers. Cell Res (2019) 29(10):832–45. doi: 10.1038/s41422-019-0225-9 PMC6796873 31481760

[42] Chen D Bhat-Nakshatri P Goswami C Badve S Nakshatri H. ANTXR1, a stem cell-enriched functional biomarker, connects collagen signaling to cancer stem-like cells and metastasis in breast cancer. Cancer Res (2013) 73(18):5821–33. doi: 10.1158/0008-5472.CAN-13-1080 PMC3778138 23832666

[43] Chen C Okita Y Watanabe Y Abe F Fikry MA Ichikawa Y. Glycoprotein nmb is exposed on the surface of dormant breast cancer cells and induces stem cell-like properties. Cancer Res (2018) 78(22):6424–35. doi: 10.1158/0008-5472.CAN-18-0599 30224376

[44] Ebinger S Ozdemir EZ Ziegenhain C Tiedt S Castro Alves C Grunert M. Characterization of rare, dormant, and therapy-resistant cells in acute lymphoblastic leukemia. Cancer Cell (2016) 30(6):849–62. doi: 10.1016/j.ccell.2016.11.002 PMC5156313 27916615

[45] Sato N Fukushima N Chang R Matsubayashi H Goggins M. Differential and epigenetic gene expression profiling identifies frequent disruption of the RELN pathway in pancreatic cancers. Gastroenterology. (2006) 130(2):548–65. doi: 10.1053/j.gastro.2005.11.008 16472607

[46] Serrano-Morales JM Vazquez-Carretero MD Peral MJ Ilundain AA Garcia-Miranda P. Reelin-Dab1 signaling system in human colorectal cancer. Mol Carcinog (2017) 56(2):712–21. doi: 10.1002/mc.22527 27434856

[47] Becker J Frohlich J Perske C Pavlakovic H Wilting J. Reelin signalling in neuroblastoma: migratory switch in metastatic stages. Int J Oncol (2012) 41(2):681–9. doi: 10.3892/ijo.2012.1488 22614235

[48] Liu Q Luo Q Halim A Song G. Targeting lipid metabolism of cancer cells: A promising therapeutic strategy for cancer. Cancer Lett (2017) 401:39–45. doi: 10.1016/j.canlet.2017.05.002 28527945

[49] Nguyen A Tao H Metrione M Hajri T. Very low density lipoprotein receptor (VLDLR) expression is a determinant factor in adipose tissue inflammation and adipocyte-macrophage interaction. J Biol Chem (2014) 289(3):1688–703. doi: 10.1074/jbc.M113.515320 PMC3894347 24293365

[50] Lu CW Lo YH Chen CH Lin CY Tsai CH Chen PJ. But not HDL, promote breast cancer cell proliferation, metastasis and angiogenesis. Cancer Lett (2017) 388:130–8. doi: 10.1016/j.canlet.2016.11.033 27940127

[51] Sundelin JP Stahlman M Lundqvist A Levin M Parini P Johansson ME. Increased expression of the very low-density lipoprotein receptor mediates lipid accumulation in clear-cell renal cell carcinoma. PloS One (2012) 7(11):e48694. doi: 10.1371/journal.pone.0048694 23185271 PMC3501495

[52] Frykman PK Brown MS Yamamoto T Goldstein JL Herz J. Normal plasma lipoproteins and fertility in gene-targeted mice homozygous for a disruption in the gene encoding very low density lipoprotein receptor. Proc Natl Acad Sci USA (1995) 92(18):8453–7. doi: 10.1073/pnas.92.18.8453 PMC41175 7667310

[53] Nakamura Y Yamamoto M Kumamaru E. Very low-density lipoprotein receptor in fetal intestine and gastric adenocarcinoma cells. Arch Pathol Lab Med (2000) 124(1):119–22. doi: 10.5858/2000-124-0119-VLDLRI 10629142

[54] Luo M Liu YJ Xia LM Yan W Zhu Q Tian DA. Very low density lipoprotein receptor subtype II silencing by RNA interference inhibits cell proliferation in hepatoma cell lines. Hepatogastroenterology (2010) 57(101):882–90. 21033246

