# Supplementary material for: VLDLR disturbs quiescence of breast cancer stem cells in a ligand-independent function
Source: Front Oncol. 2022 Dec 7;12:887035. doi: 10.3389/fonc.2022.887035 (PMC9767959; doi:10.3389/fonc.2022.887035)
Supplement: Supplementary file 1 [file DataSheet_1.docx]

Supplementary Material

#
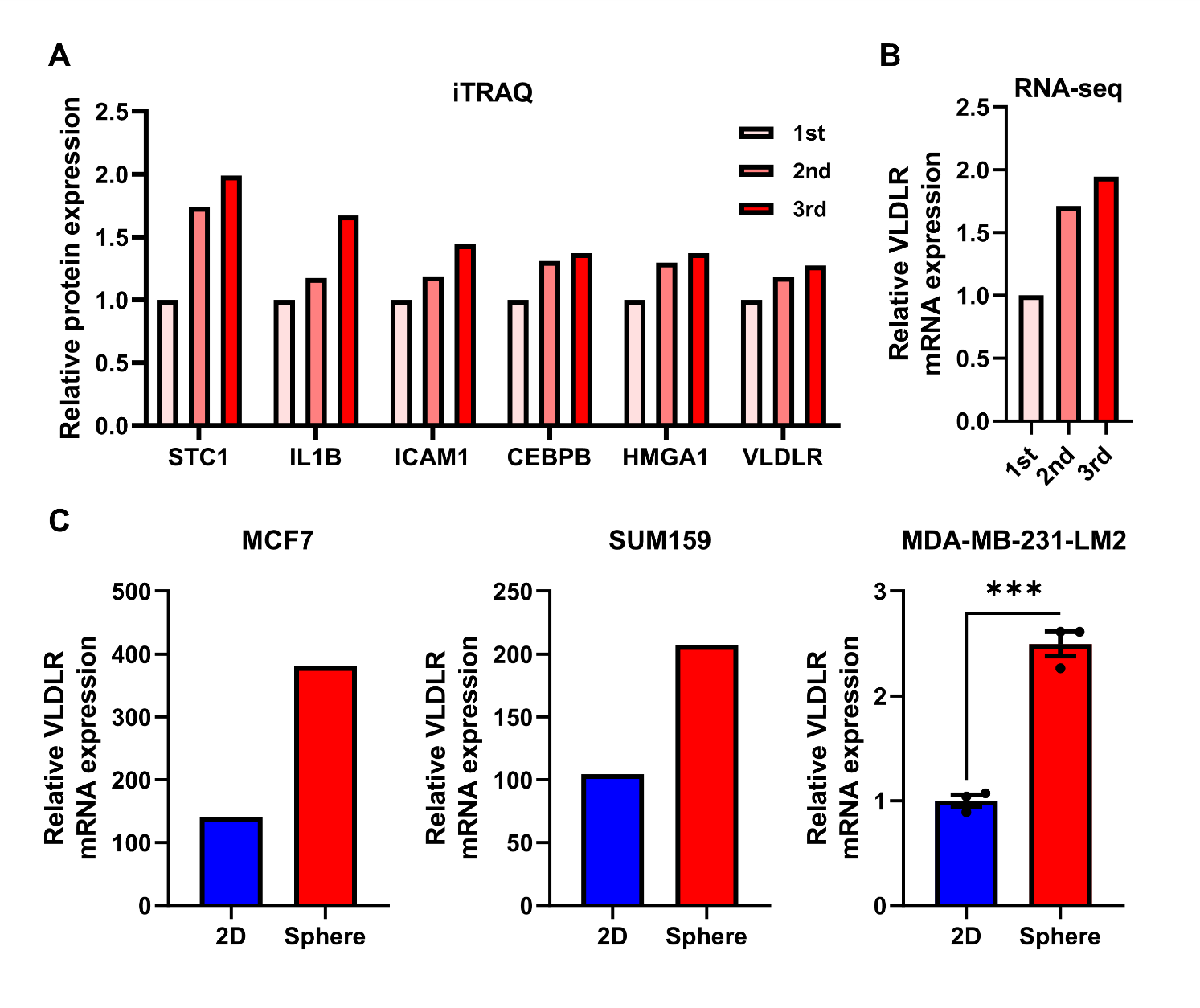


**Supplementary Figure 1. VLDLR is elevated in BCSC population. (A, B)** BCSCs in MDA-MB-231 cells were enriched by serial sphere formation assay. The expression levels of indicated proteins **(A)** and VLDLR mRNA **(B)** were determined by iTRAQ-MS and RNA-seq, respectively. **(C)** Gene expression of VLDLR was compared between monolayer cells (2D) and sphere-forming cells (Sphere) derived from MCF7, SUM159 and MDA-MB-231-LM2 cells using genome mRNA expression profiling data (GSE43657 and GSE98239) from GEO database. Data of MDA-MB-231-LM2 cells are presented as the mean ± S.E.M.. The unpaired t test was used for statistical analysis, ⁎⁎⁎*p* < 0.001.


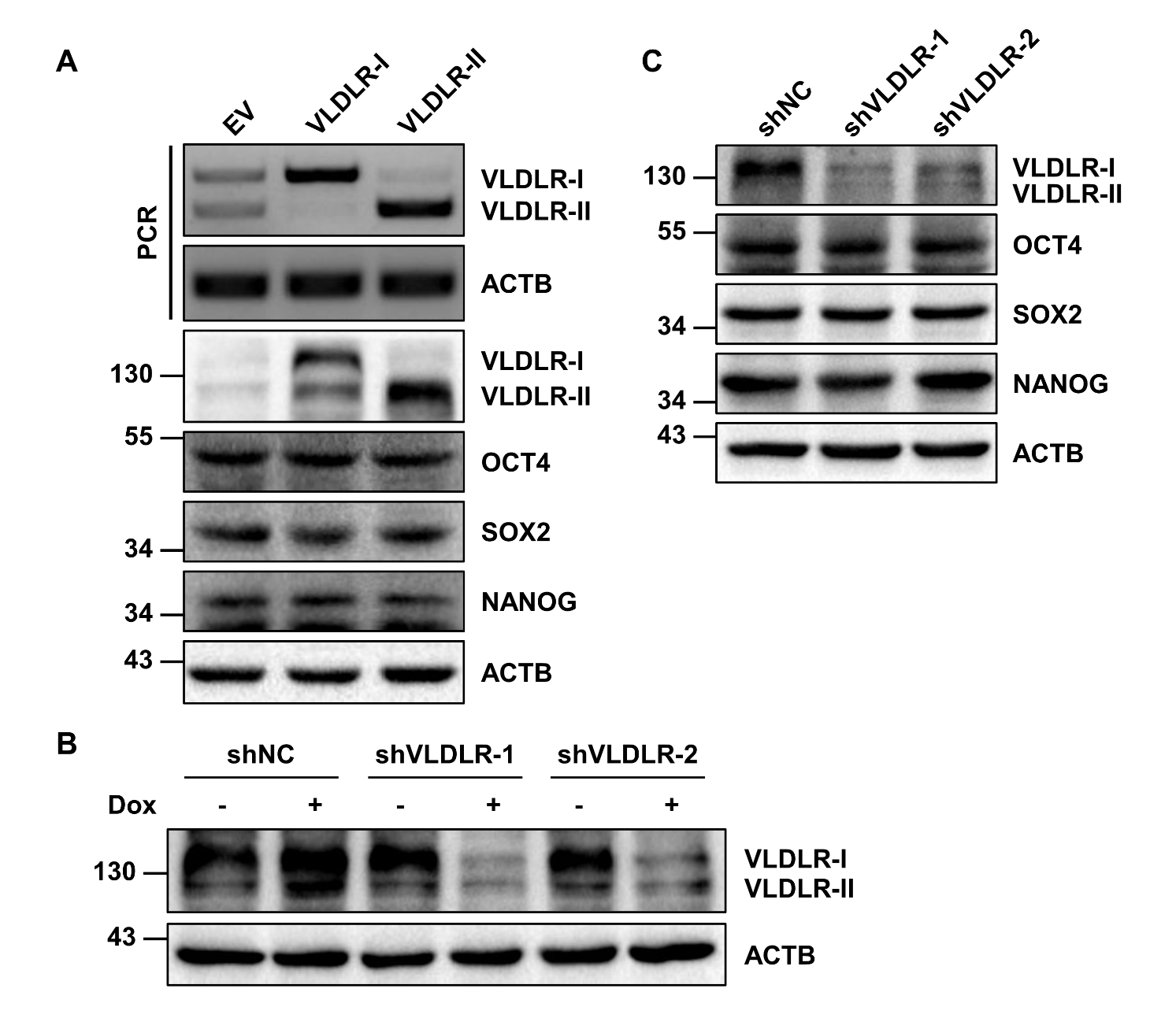


**Supplementary Figure 2. VLDLR has no detectable effects on the expression of OCT4, SOX2 and NANOG. (A)** Efficiencies of VLDLR-I/II overexpression in MDA-MB-231 cells were examined by reverse-transcriptase polymerase chain reaction (RT–PCR) and western blot analysis. The expression levels of stemness-related proteins (OCT4, SOX2, NANOG) were analyzed by western blot. ACTB was used as the internal control. EV: empty vector. **(B)** Efficiencies of shRNAs-mediated VLDLR knockdown in MDA-MB-231 cells with or without doxycycline (Dox) treatment (2 μg/mL, 5 days) were examined by western blot analysis. ACTB was used as the internal control. **(C)** The expression levels of stemness-related proteins (OCT4, SOX2, NANOG) in MDA-MB-231 cells were analyzed by western blot upon VLDLR knockdown for 5 days. ACTB was used as the internal control.


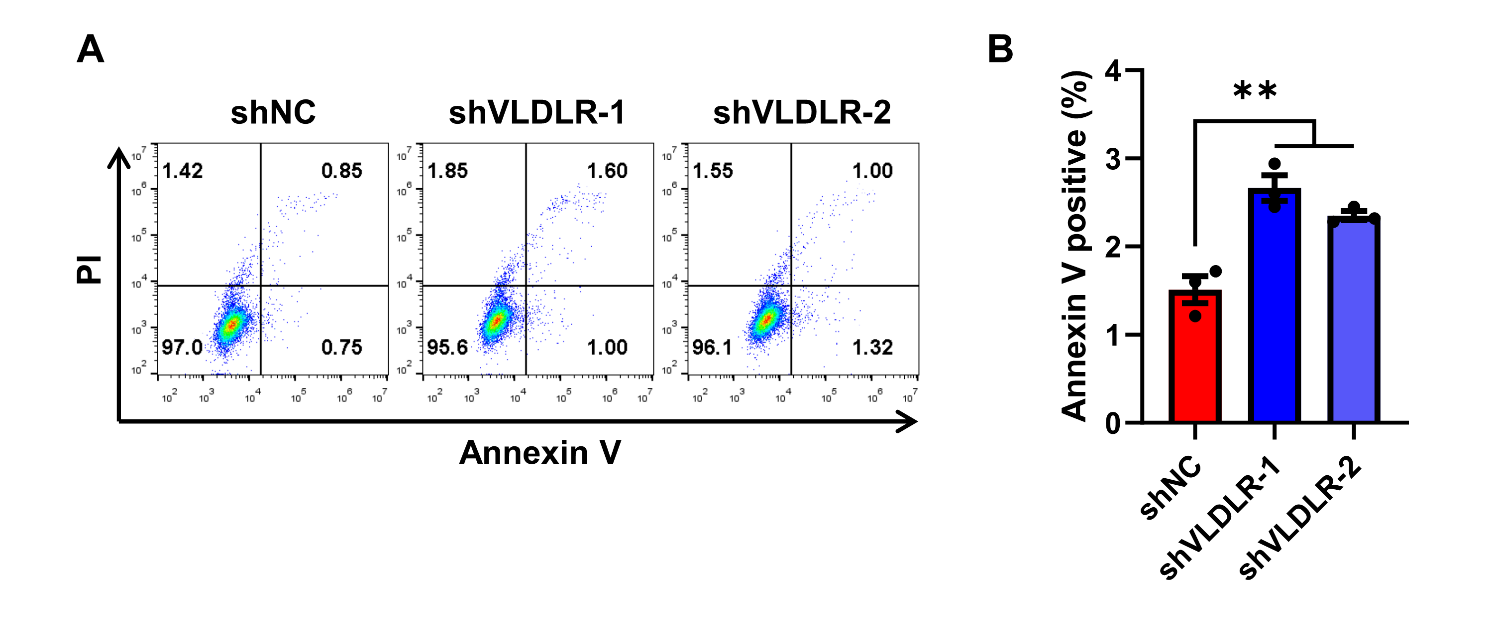


**Supplementary Figure 3. The percentages of apoptotic cells were slightly increased upon VLDLR silencing. (A, B)** Cellular apoptosis of control (shNC) and VLDLR knockdown cells were measured by Annexin V/PI staining and analyzed by flow cytometry. Data are presented as the mean ± S.E.M. of three independent experiments. One-way ANOVA followed by Dunnett’s multiple comparisons test was used for statistical analysis, ⁎⁎*p* < 0.01.


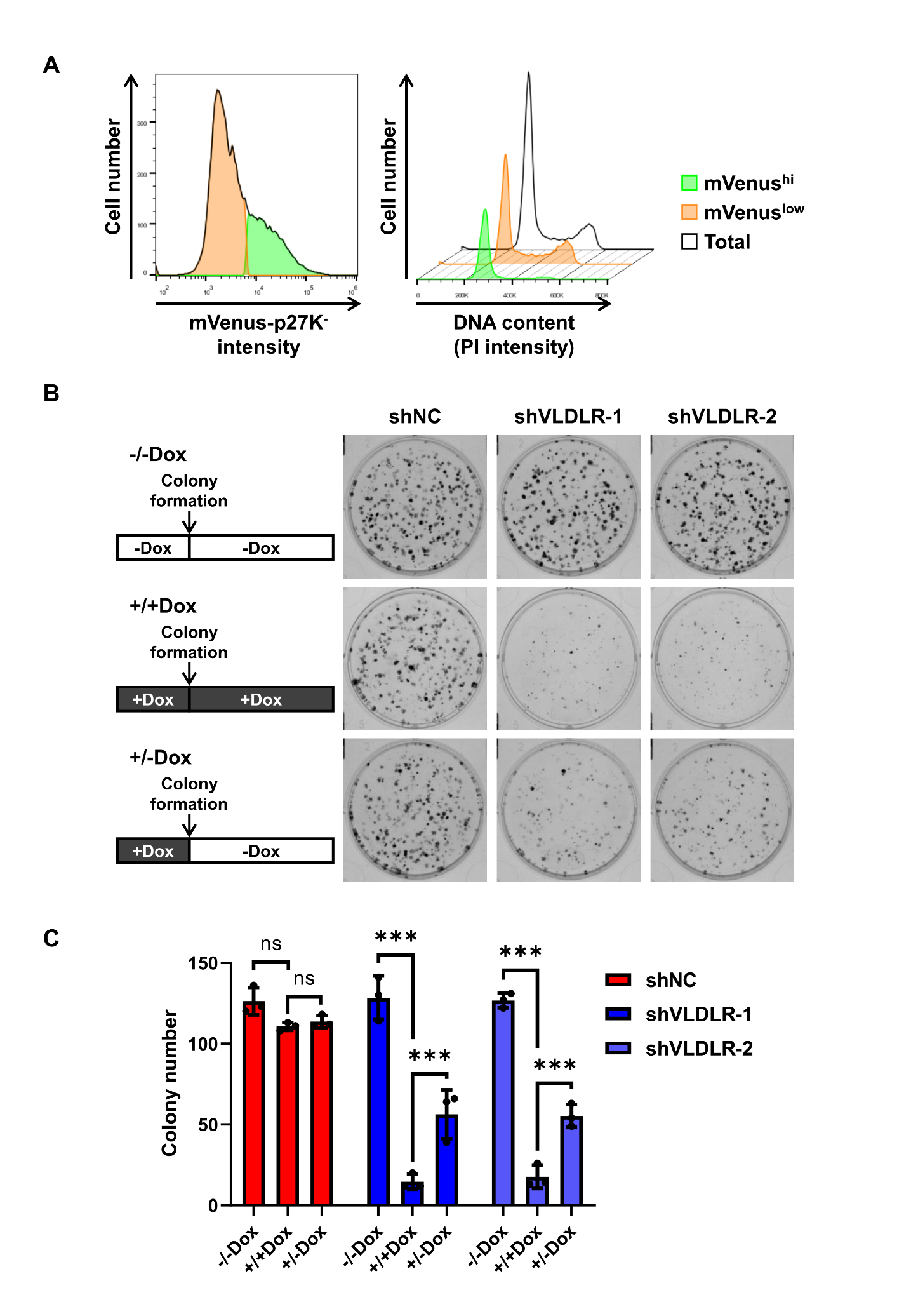


**Supplementary Figure 4. Transition to quiescence is induced by VLDLR silencing. (A)** The mVenus fluorescent intensity can be used as an indicator of quiescence in the stable cell line expressing mVenus-p27K^-^ fusion protein. The mVenus fluorescent intensity and cell cycle profile of this stable cell line were analyzed by ﬂow cytometry. **(B, C)** MDA-MB-231 cells with undisturbed VLDLR expression (-/-Dox, without Dox treatment), silenced VLDLR expression (+/+Dox, continuous Dox treatment), and restored VLDLR expression (+/-Dox, Dox withdraw) were subjected to colony formation assay. Cells were stained with crystal violet and representative photographs were shown **(B)**. The colony numbers were counted with ImageJ **(C)**. Data are presented as the mean ± S.E.M. of three independent experiments. One-way ANOVA followed by Dunnett’s test was used for multiple group comparison. ns: no significance, ⁎⁎⁎*p* < 0.001.


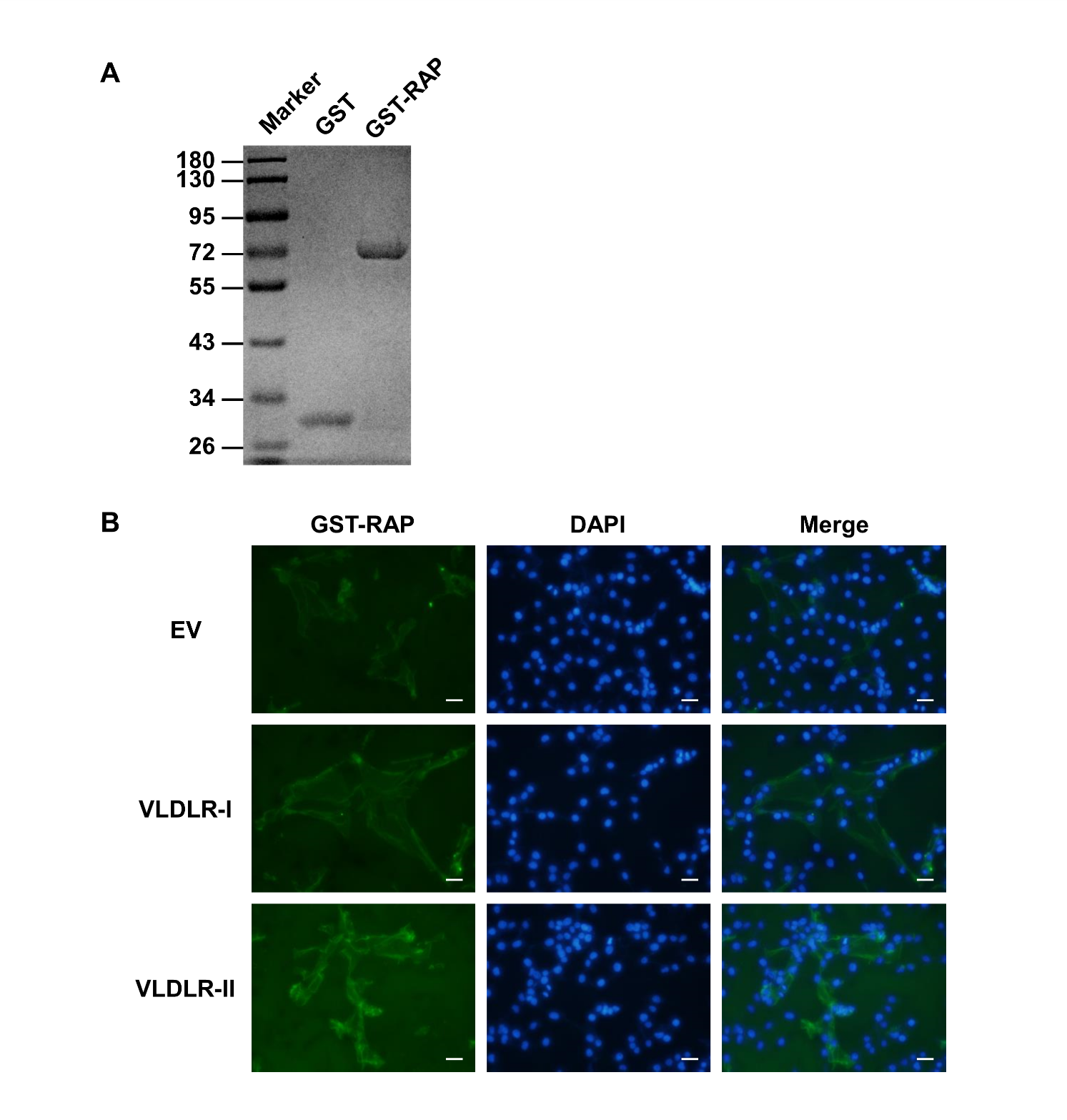


**Supplementary Figure 5. GST-RAP fusion protein can bind to cell membrane. (A)** Purified GST and GST-RAP fusion protein were analyzed using SDS–PAGE followed by Coomassie blue staining. **(B)** Control (EV) and VLDLR-I/II overexpressed cells were treated for 72 hours with 200 nM GST-RAP fusion protein. The cell membrane bound GST-RAP were observed with immunofluorescent staining. Representative images were shown. Scale bar: 50 μm. EV: empty vector.


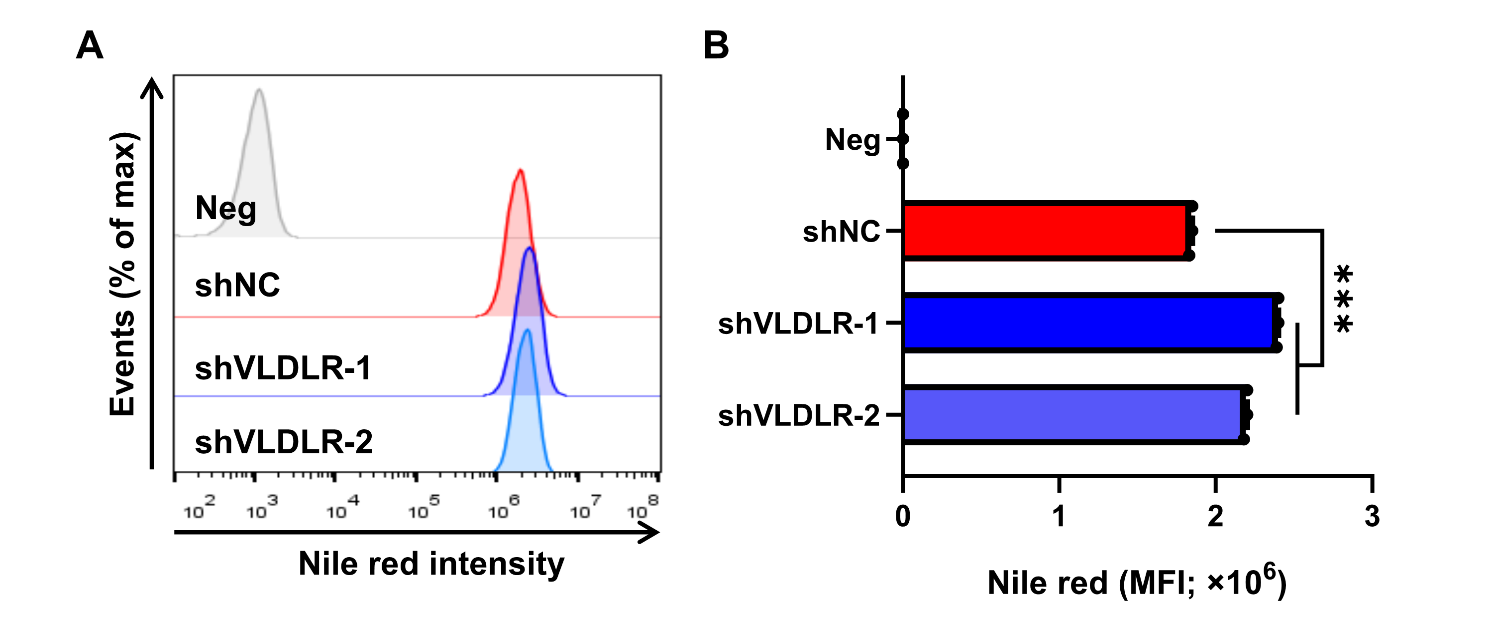


**Supplementary Figure 6. VLDLR knockdown did not inhibit lipid accumulation in breast cancer cells. (A, B)** Control (shNC) and VLDLR knockdown cells were stained with Nile red and analyzed by flow cytometry. Negative control was performed by incubating the cells without Nile red. MFI, Mean Fluorescence Intensity. Data are presented as the mean ± S.E.M. of three independent experiments. One-way ANOVA followed by Dunnett’s multiple comparisons test was used for statistical analysis, ⁎⁎⁎*p* < 0.001.


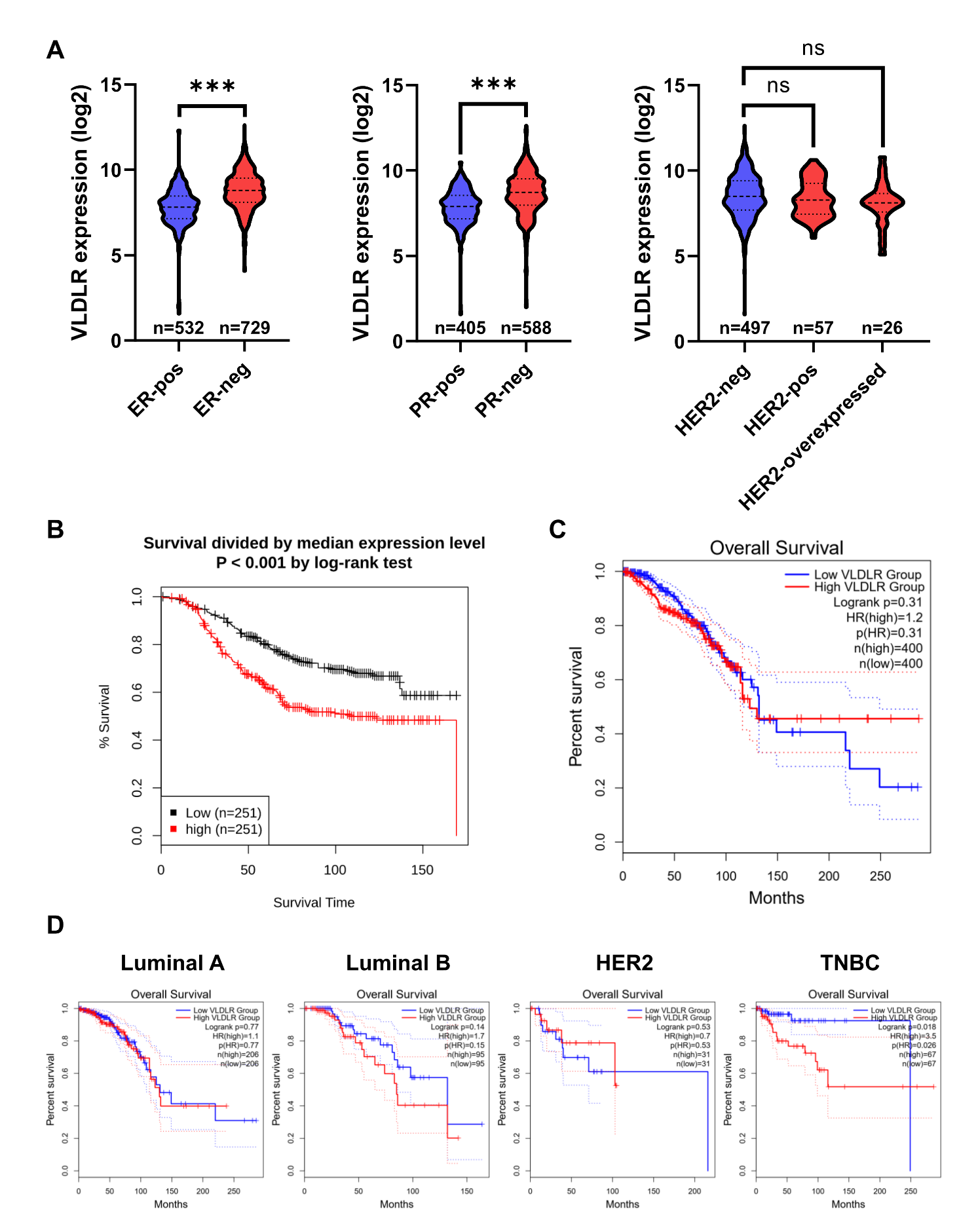


**Supplementary Figure 7. Elevated VLDLR expression predicts poor prognosis of breast cancer. (A)** The VLDLR mRNA expression was analyzed in breast cancer tissues with different status of ER, PR and HER2 expression using GENT2 database. The unpaired t test (left and middle panel) and one-way ANOVA followed by Dunnett’s multiple comparisons test (right panel) was used for statistical analysis. ns: no significance, ⁎⁎⁎*p* < 0.001. **(B, C)** Overall survival of breast cancer patients with high or low expression of VLDLR was analyzed using GENT2 **(B)** and GEPIA2 database **(C)**. **(D)** Overall survival of breast cancer patients with high or low expression of VLDLR was analyzed in each indicated breast cancer subtype using GEPIA2 database.
